# Targeting FLT3-TAZ signaling to suppress drug resistance in blast phase chronic myeloid leukemia

**DOI:** 10.1186/s12943-023-01837-4

**Published:** 2023-11-06

**Authors:** Ji Eun Shin, Soo-Hyun Kim, Mingyu Kong, Hwa-Ryeon Kim, Sungmin Yoon, Kyung-Mi Kee, Jung Ah Kim, Dong Hyeon Kim, So Yeon Park, Jae Hyung Park, Hongtae Kim, Kyoung Tai No, Han-Woong Lee, Heon Yung Gee, Seunghee Hong, Kun-Liang Guan, Jae-Seok Roe, Hyunbeom Lee, Dong-Wook Kim, Hyun Woo Park

**Affiliations:** 1 Department of Biochemistry, College of Life Science and Biotechnology, Yonsei University, Seoul, 03722 Republic of Korea; 2 Leukemia Omics Research Institute, Eulji University, Uijeongbu-si, Gyeonggi-Do Republic of Korea; 3 Center for Advanced Biomolecular Recognition, Korea Institute of Science and Technology, Seoul, 02792 Korea; 4 Department of Pharmacology, Graduate School of Medical Science, Brain Korea 21 Project, Yonsei University College of Medicine, Seoul, 03722 Republic of Korea; 5 School of Life Sciences, Ulsan National Institute of Science and Technology, Ulsan, Republic of Korea; 6 Department of Biotechnology, College of Life Science and Biotechnology, Yonsei University, Seoul, 03722 Republic of Korea; 7 Bioinformatics and Molecular Design Research Center (BMDRC), Incheon, 21983 Korea; 8 Department of Pharmacology and Moores Cancer Center, University of California San Diego, La Jolla, CA 92093 USA; 9 Hematology Department, Eulji Medical Center, Eulji University, Uijeongbu-si, Gyeonggi-Do Republic of Korea

**Keywords:** FLT3, Drug resistance, Hippo-YAP/TAZ pathway, Blast phase, Ponatinib, Midostaurin, CML, AML, Cancer, CD36

## Abstract

**Background:**

Although the development of BCR::ABL1 tyrosine kinase inhibitors (TKIs) rendered chronic myeloid leukemia (CML) a manageable condition, acquisition of drug resistance during blast phase (BP) progression remains a critical challenge. Here, we reposition FLT3, one of the most frequently mutated drivers of acute myeloid leukemia (AML), as a prognostic marker and therapeutic target of BP-CML.

**Methods:**

We generated FLT3 expressing BCR::ABL1 TKI-resistant CML cells and enrolled phase-specific CML patient cohort to obtain unpaired and paired serial specimens and verify the role of FLT3 signaling in BP-CML patients. We performed multi-omics approaches in animal and patient studies to demonstrate the clinical feasibility of FLT3 as a viable target of BP-CML by establishing the (1) molecular mechanisms of FLT3-driven drug resistance, (2) diagnostic methods of FLT3 protein expression and localization, (3) association between FLT3 signaling and CML prognosis, and (4) therapeutic strategies to tackle FLT3^+^ CML patients.

**Results:**

We reposition the significance of FLT3 in the acquisition of drug resistance in BP-CML, thereby, newly classify a FLT3^+^ BP-CML subgroup. Mechanistically, FLT3 expression in CML cells activated the FLT3-JAK-STAT3-TAZ-TEAD-CD36 signaling pathway, which conferred resistance to a wide range of BCR::ABL1 TKIs that was independent of recurrent BCR::ABL1 mutations. Notably, FLT3^+^ BP-CML patients had significantly less favorable prognosis than FLT3^−^ patients. Remarkably, we demonstrate that repurposing FLT3 inhibitors combined with BCR::ABL1 targeted therapies or the single treatment with ponatinib alone can overcome drug resistance and promote BP-CML cell death in patient-derived FLT3^+^ BCR::ABL1 cells and mouse xenograft models.

**Conclusion:**

Here, we reposition FLT3 as a critical determinant of CML progression via FLT3-JAK-STAT3-TAZ-TEAD-CD36 signaling pathway that promotes TKI resistance and predicts worse prognosis in BP-CML patients. Our findings open novel therapeutic opportunities that exploit the undescribed link between distinct types of malignancies.

**Graphical Abstract:**

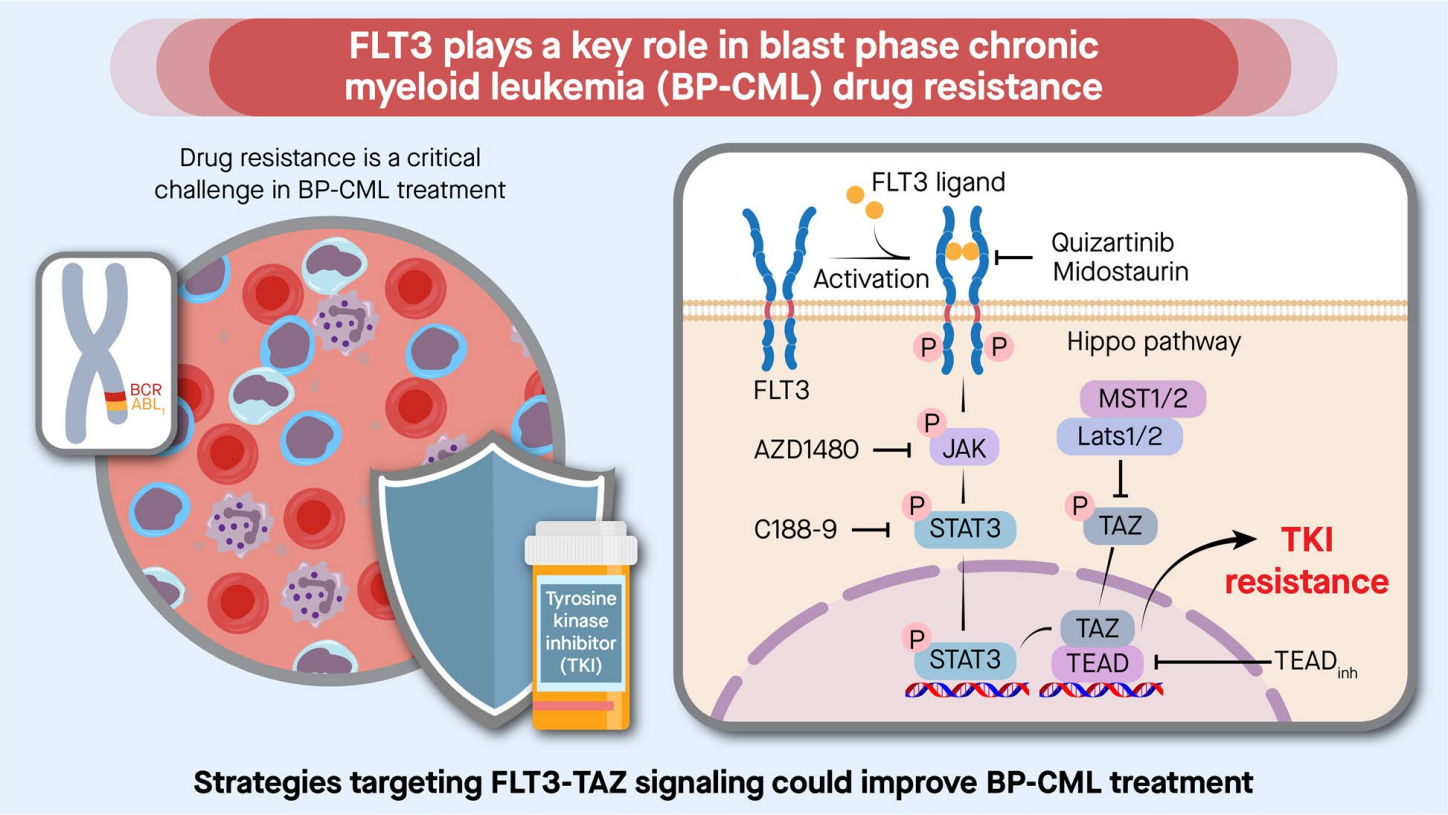

**Supplementary Information:**

The online version contains supplementary material available at 10.1186/s12943-023-01837-4.

## Introduction

Chronic myeloid leukemia (CML) is characterized by a massive expansion of predominately myeloid lineage cells driven by the constitutively active BCR::ABL1 fusion tyrosine kinase. Although most patients with CML are diagnosed in the chronic phase (CP) and achieve long-term disease control, subset of patients would progress into advanced phases of CML, which are referred to as accelerated phase (AP) or blast phase (BP). These are defined by a substantial increase in blast count (immature blood cells) that phenotypically resembles acute leukemia [1, 2]. Unfortunately, while outcomes for patients with CML improved greatly with the development of BCR::ABL1 TKIs, CML-related deaths due to progression from chronic to advanced disease occur in up to 15% of patients with limited therapy options depending on clinical factors present at diagnosis and types of TKI treatment [3–5].

Responses to TKIs are short-lived in the advanced phases of CML due to drug resistance and relapses driven by both BCR::ABL1 kinase-dependent and -independent mechanisms [6]. BCR::ABL1 kinase-dependent drug resistance often coincides with the acquisition of point mutations within the BCR::ABL1 kinase domain that impair drug binding, thereby restoring constitutive tyrosine kinase activity. Several successive generations of TKIs have been approved in an effort to address the BCR::ABL1 point mutation resistance vulnerabilities of CML patients, however, predicting BP progression and effectively treating BP patients with resistance remains an area of clinical challenge. It is therefore critical to further exploit strategies that target the BCR::ABL1 kinase-independent mechanisms that contribute to TKI resistance through reactivation of critical signaling pathways [6, 7].

It is interesting that although the evolution of CML from CP to BP phenotypically resembles acute myelogenous leukemia (AML), the recommended treatments for BP-CML and AML patients are completely different because the two diseases have distinct driver genes [2]. Recent years have seen numerous advancements in AML therapy, including genetically targeted therapies against FLT3, IDH1/2, and TP53 [8, 9]. FLT3 is one of the most frequently mutated genes associated with AML [10]. The FLT3 inhibitors midostaurin and gilteritinib were recently approved and several new FLT3 inhibitors are currently in clinical trials. Hence, we investigated whether genes and pathways involved in AML are dysregulated in advanced phases of CML, leading to the acquisition of drug resistance to BCR::ABL1 TKIs.

Here, we report our discovery that aberrant FLT3 induction activates FLT3-JAK/STAT3-TAZ-TEAD-CD36 signaling pathway, which confers drug resistance to a wide range of BCR::ABL1 TKIs. From unpaired and paired BP-CML patient cohorts, we found that FLT3-TAZ signaling was largely absent in CP samples but present in approximately half of the BP-CML samples. In contrast to FLT3^−^ BP-CML patients, FLT3^+^ BP-CML patients showed strong correlations with the prognostic factors of CML and were associated with less favorable prognosis. Based on these results, we have demonstrated that co-administration of BCR::ABL1 inhibitors with recently developed FLT3 inhibitors, such as midostaurin or quizartinib, suppressed FLT3-TAZ signaling pathway, restored TKI sensitivity, and promoted cell death in FLT3^+^ BP-CML cells both in vitro and in vivo. Moreover, single treatment with ponatinib, a third generation BCR::ABL1 TKI that targets BCR::ABL1 and FLT3 simultaneously, recapitulated the inhibitory mechanism and therapeutic efficacy of combined treatments against drug resistant FLT3^+^ BCR::ABL1 cells. Together, our findings demonstrate that FLT3 can be used as a prognostic marker and therapeutic target in BP progression and propose that targeting FLT3 and downstream effectors are viable treatment strategies for preventing and overcoming TKI resistance in advanced phase CML.

## Methods

### Public data analysis

The DNA microarray data of CML patients used in this study were retrieved from the Gene Expression Omnibus. The mRNA expression data of CML patients in the chronic phase (CP), accelerated phase (AP), and blast phase (BP) were obtained from GSE4170. YAP/TAZ transcript expression data were obtained from the Human Protein Atlas (HPA) database in the form of matrices that included normalized transcripts per million (nTPM) values. p-values were calculated using Prism 9.

### Cell culture, viral infection, and generation of TKI-resistance K562 cells

K562 (ATCC, CCL-243) and K562-IMR-ATCC (spontaneous imatinib-resistant cell line), (ATCC, CCL-3344), AR230 (ATCC, CRL-3345) cells were purchased from the American Type Culture Collection (ATCC). K562 and AR230 cells were maintained in RPMI (Hyclone, SH30027.01), and HEK293T cells were maintained in DMEM (Hyclone, SH30022.01) containing 10% FBS (Gibco, 16000-044) and 1% Penicillin (Gibco, 15140-122) in a humidified incubator at 37°C with 5% CO_2_. For generating spontaneous imatinib-resistant K562 cells (K562-IMR), we treated imatinib by gradually increasing the dose from 0.2 μM to 1 μM during a 6 month period. For viral infection of K562 cells and AR230, HEK293T cells were transfected with retroviral MSCV vectors containing either FLT3 constructs, p-BABE vector containing TAZ, TAZ-4SA constructs, or a lentiviral pLKO vector containing shTAZ, shTEAD constructs using transfection Reagent (Polyplus) according to the manufacturer's protocol. For generating MSCV-FLT3 plasmid, we purchased FLT3-ITD vector (Addgene, #74499) and replaced FLT3-ITD to FLT3 wild type sequence by mutagenesis. 48 hrs after transfection, the retroviral/lentiviral supernatant was filtered through a 0.45 μm filter, supplemented with 10 μm/ml polybrene, and used to infect K562 cells. 48 hrs after infection, K562 and AR230 cells were selected with puromycin (2 μm/ml). To generate TKI-resistant K562-FLT3-IR, -NR, and -DR cells, each group was treated with 1 μM imatinib, 20 nM nilotinib, and 1 μM dasatinib until the acquisition of drug resistance (about 17 days). To generate TKI-resistant AR230-FLT3-IR cells, AR230-FLT3 cells were treated with 1 μM imatinib and FL 10ng/ml until the acquisition of drug resistance (about 40 days). To maintain TKI resistance, each cell line was further cultured in media containing each drug every 3–5 days.

### Chemical compounds

The following chemical compounds were used in this study: Imatinib (Selleckchem, S24750), Dasatinib (Santacruz, sc-358114), Nilotinib (Selleckchem, S1033), Ponatinib (Selleckchem, S1490), Quizartinib (Selleckchem, S1526), Midostaurin (Cayman, 10459), AZD-1480 (Selleckchem, S2162), C188-9 (Selleckchem, S8605), BP-1-102 (Selleckchem, S7769), Ruxolitinib (Selleckchem, S1378) and FLT3 ligand (PeproTech, 30019).

### Cell growth analyses and cell viability assays

For cell growth analysis, 0.1 - 2 x 10^5^ cells were plated per 12-well plate (Falcon, 353043) with media. Each well was resuspended by pipetting and trypan blue stained cells were counted by Countess (Thermo, A27977). Cells were replaced with fresh medium containing each drug every 3–5 days. For cell viability assay, cells from each well were precipitated and assessed with the MTT assay kit (Roche, 11465007001) or the Cell Titer Glo assay kit (Promega, G9242) according to the manufacturer´s recommendations.

### Flow cytometry for cell cycle analysis and dilution assays

For each cell cycle analysis, 10^6^ cells were harvested and FxCycle™ PI/RNase Staining Solution (Thermo, F10797) was used according to the manufacturer’s protocol and added to the flow cytometer (Thermo, Attune NxT). For dilution analyses, K562-mock and K562-FLT3 cells were mixed in different ratios (1:1, 1:10, 1:100). A total of 2x10^5^ cells were treated with 1 μM imatinib for 39 days and fresh medium containing the drug was replaced every 3–5 days. On day 39, the cells were fixed in 4% paraformaldehyde (Thermo, 28908)/PBS for 15 min and then permeabilized with 0.1% Triton-X/PBS for 10 min. The cells were washed and blocked with 3% BSA/PBS for 30 minutes and then incubated for 1 hour at RT with primary antibodies diluted in 0.5% BSA/PBS followed by a 30-min incubation with secondary antibodies diluted in 0.5% BSA/PBS. Finally the cells were resuspended in PBS and analyzed by flow cytometry (Thermo, Attune NxT).

### Immunoprecipitation assay

1x10^7^ cells were rinsed once with PBS, they were in lysed with ice-cold lysis buffer (0.15 M NaCl, 0.05 M Tris-HCl, 0.5% Triton X-100), and one tablet each of EDTA-free protease/phosphatase inhibitor cocktail (Thermo, 78446). For immunoprecipitations, magnetic beads (Bio-rad, 1614023) and TEAD antibody (Santa Cruz, sc-101184) were added to the lysates and incubated overnight at 4°C. Then, the samples were washed with lysis buffer, denatured with the addition of 2X sample buffer, boiled for 8 min and subjected to immunoblotting analysis.

### Immunofluorescence microscopy

Cells were seeded onto 24-well plates on coverslips (Everest, SB-Shifix25) and incubated for 30 min. The cells were fixed with 4% paraformaldehyde (Thermo, 28908) in PBS for 20 min and then permeabilized with 0.1% Triton-X/PBS for 10 min. After cells were washed and blocked with 3% BSA/PBS for 30 minutes, they were incubated overnight at 4°C with primary antibodies diluted in 3% BSA/PBS. Secondary antibodies were diluted in 3% BSA/PBS. Then, the cells were incubated for 2 hrs with secondary antibodies diluted in 3% BSA/PBS. Finally, the slides were mounted with Prolong gold antifade reagent containing DAPI (Invitrogen, P36930), and fluorescent images were acquired using a confocal microscope.

### RNA extraction, cDNA synthesis, and quantitative real-time PCR analysis

After harvesting 2 x 10^5^ cells for RNA extraction using the RNeasy Plus mini kit (Qiagen, 4134), the resulting RNA samples were reverse transcribed to complementary DNA using iScript reverse transcriptase (Bio-Rad, 1708890). Then, qRT-PCR was performed using the KAPA SYBR FAST Kapa SYBR FAST qPCR Master Mix (KM4103) and the 4376600 real-time PCR system (Applied Biosystems). The following primers were used for qPCR : FLT3, 5’- AAGCAATTTAGGTATGAAAGCCAGC-3’ (forward) and 5’-CTTTCAGCATTTTGACGGCAACC-3’ (reverse); YAP, 5’-CCTTCTTCAAGCCGCCGGAG-3’ (forward) and 5’-CAGTGTCCCAGGAGAAACAGC-3’ (reverse); TAZ, 5’-AATGGAGGGCCATATCATTCGAG-3’ (forward) and 5’-GTCCTGCGTTTTCTCCTGTATC- 3’ (reverse); CD36, 5’-CAGGTCAACCTATTGGTCAAGCCT-3’ (forward) and 5’-GCCTTCTCATCACCAATGGTCC-3’ (reverse); GAPDH, 5’-GCAAATTCCATGGCACCGT-3’ (forward) and 5’-TCGCCCCACTTGATTTTGG-3’ (reverse); HPRT, 5’-AGAATGTCTTGATTGTGGAAGA-3’ (forward) and 5’-ACCTTGACCATCTTTGGATTA-3’ (reverse) for human samples. mFLT3, 5’- GTGCTGACGTTTGAAGACCTCC-3’ (forward) and 5’-GGTGACCAACACATTCCTGGCT-3’ (reverse); mHPRT, 5’-GCAGTACAGCCCCAAAATGG-3’ (forward) and 5’-ACAAAGTCCGGCCTGTATCCAA-3’ (reverse) for mouse samples.

### Immunoblotting analysis

After precipitating cells by centrifugation at 3000 g for 3 min and lysing them in SDS sample buffer, the resulting protein samples were loaded onto the wells of a 9% SDS-page gel and transferred to PVDF membranes (Millipore). The membranes were blocked with 5% skim milk in TBS-T buffer (trisbuffered saline with 0.2% Tween-20) for 1 hr at room temperature before incubation with the corresponding primary antibodies overnight at 4˚C with gentle agitation. Then, after the membranes were washed 3 times with TBS-T buffer, they were incubated with secondary antibody in 5% skim milk/TBS-T. After the membranes were washed 4 times, they were developed using X-ray films.

The antibodies used in this study are as follows: Vinculin (Santacruz, sc-7364), BCR::ABL1 (CST, 2862), CD36 (CST, 14347), GAPDH (Santacruz, sc-25778), FLT3 (CST, 3462), p-FLT3 Y842 (CST, 4577), p-FLT3 Y591 (CST, 3474), p-STAT3 (CST, 9145), p-STAT5 (CST, 9351), p-STAT1(CST, 9167), STAT1 (CST, 9172), STAT5 (CST, 25656), pan-TEAD (CST, 13295), STAT3 (CST, 4904), TAZ (CST, 83669), YAP (63.7) (Santcruz, sc-101199), YAP/TAZ (CST, 8418), LATS (CST, 3477), PARP (CST, 9532), p-BCR::ABL1 Y412 (CST, 2865), p-BCR::ABL1 Y245 (CST, 2861), p-BCR Y177 (CST, 3901), p-ERK (CST, 4377), ERK 1/2 (CST, 4695), p-S6K (CST, 9234), p-Src (CST, 2101), p-AKT (CST, 4060), p-P65 (CST, 3033), b-Actin (Santacruz, sc-47778), MEK1/2 (CST, 8727), Histone H3 (CST, 4499), and Lamin A/C (CST, 4777).

### Colony formation assay

Each well of a 6-well plate (Falcon, 353046) was coated with 1.5 ml of bottom agar (RPMI containing 10% FBS and 0.8% low melting agarose (Sigma, A9414). Then, 1–2 x 10^4^ cells were suspended in 1.5 ml of top agar (RPMI containing 10% FBS and 0.4% low melting agarose) and plated into each well. The cells were incubated for approximately three weeks. Fresh media exchanges containing each drug were performed every three days. Colonies were stained using 0.005% crystal violet and the colonies per well were counted.

### siRNA transfection

K562-FLT3-IR cells were transfected with transfection mix (100 μl of Opti-MEM™ medium (ThermoFisher, #31985070), 12 μl RNAi Max (Invitrogen, 13778150), and 2 μl of 20 μM siRNA) for 48 hrs. The Neon Transfection System (ThermoFisher, MPK5000) was used to electroporate the K562-mock, -IMR, and -FLT3-IR cells for the purpose of introducing siRNA molecules (ABL siRNA). The conditions of electroporation for this study: 1450V, 3 pulse, 10ms, 50 nM DNA, and 1x10^5^ cells. The siRNA used in this study are as follows: control siRNA (Dharmacon siGENOME non-targeting control pool #D-001206-13-200) and siLATS1/2 (Dharmacon siGENOME SMARTPool #M-004632-00-0005 and #M-003865-02-0005), and siABL (Dharmacon, #L-003100-00-0010).

### Metabolites profiling using UPLC-Orbitrap-MS

#### Sample preparation

After adding 100 μL of ice-cold 70% methanol containing an internal standard (reserpine at a final concentration of 2 ppm) to the cell pellets (3 x 10^6^), the solution was vortexed for 30 sec. Then, liquid nitrogen was used to perform three successive freeze/thaw cycles to lyse the cells. The cell lysates were centrifuged for 10 min at 14,000 rpm. The supernatants were immediately used in metabolomic experiments. An equal volume of each sample was pooled to create a quality control (QC) sample. A normalization of the DNA was then performed on the lysates and the concentrations were measured with a Nano-MD UV-Vis spectrophotometer (Scinco, Seoul, Korea).

#### Instrumental conditions

An Acquity® UPLC HSS T3 column (1.8 μm particle size, 2.1 mm x 100 mm, Waters, USA) was used to perform liquid chromatography on an Ultimate 3000 UHPLC system (Thermo Scientific, Jose, CA, USA) at 40°C. Mobile phase A (0.1% formic acid in distilled water) and mobile phase B (0.1% formic acid in methanol) were combined and set to flow at 0.4 mL·min-1 for the gradient elution. The sample analysis was performed using a gradient program as follows: the initial conditions, 99% A and 1% B (v/v), were maintained for 1 min, and then a linear gradient was initiated that reached 20% B over 2 min. After an increase to 70% mobile phase B for 4 min, it was slowly increased to 100% over 6 min and maintained at 100% for 2.5 min. The column was re-equilibrated to the initial conditions within 1.5 min and stabilized for 2 min. The injection volume was 10 μL and all samples were maintained at 5°C during the analysis. Prior to running the sample sequence, the QC sample was examined for conditioning. It was also examined in the first, middle, and final samples of the batch to assess instrument consistency. An LTQ Orbitrap Velos Pro™ system mass spectrometer (Thermo Scientific, San Jose, CA, USA) equipped with a heated electrospray ionization source (HESI) in positive and negative ionization mode was used for the detection of metabolites. The HESI parameters were as follows: heater temperature, 200°C; sheath gas flowrate, 35 arb (arbitrary units); auxiliary gas flow rate, 5 arb; sweep gas flowrate, 10 arb; capillary temperature, 320°C; and S-lens RF level, 67.5%. Data acquisition was performed at a resolution of 60,000 in the centroid mode using a mass range of m/z 50–1000. The Xcalibur 2.2 (Thermo Scientific, San Jose, CA, USA) software system was used for data acquisition and processing.

#### Statistical analysis and metabolite identification

Peak alignments and data collection were carried out using Xcalibur 2.2 and Compound Discoverer 2.1 (Thermo Fisher Scientific, San Jose, CA, USA). Prior to statistical analysis, the compound intensities were normalized to internal standard areas. SIMCA 14.1 (Umetrics, Inc., Ume, Sweden) and Metabolanalyst (http://www.metaboanalyst.ca/) were used to perform the multivariate analysis. Meanwhile, the variable importance in the projection (VIP) values that suggested greater discriminatory power for each metabolite were taken as the coefficients for peak selection. The Student’s t-test was used to identify statistically significant metabolites among the different groups.The HMDB, Metlin, and Lipidblast databases were used to perform putative identification of the metabolites via MS/MS fragmentation matching.

### RNA sequencing analysis

For the RNA-seq experiment, two independent experiments were performed in triplicate and pooled for RNA extraction. Raw single-strand sequencing data were aligned with the Salmon package and mapped to the human GRCh38 reference genome. Ensembl gene IDs were annotated with their respective gene symbols using the bioMart package. Reads per kilobase of transcript per million mapped reads (RPKM) were calculated for each gene. Batch correction was done using ‘removeBatchEffect’ function in the Limma R package. PCA analysis was performed using the plotMDS package, and analysis of differentially expressed genes was done using the Limma R package. The RNA-seq data reported in this study are available in the Gene Expression Omnibus (GEO) database under GSE226360.

#### Public RNA sequencing data analysis

To investigate differences based on FLT3 induction, we performed a bulk RNA-seq data analysis using the Limma R package. This analysis was independent of the experimental data analysis and focused specifically on comparing gene expression profiles between samples with FLT3 induction and those without. For this analysis, we utilized public bulk RNA-seq data (Accession no. GSE4170) as the control group. The same analysis pipeline based on the Limma R package was applied to compare the gene expression profiles between the FLT3 induced K562 cells with WT K562 cells.

### Chromatin immunoprecipitation (ChIP) assay

K562 or HEK293A cells were cross-linked for 15 min with 1% formaldehyde and then quenched with 0.125 M glycine for 10 min at room temperature. 5 × 10^6^ cells were used for H3K27ac ChIP, and 3 × 10^7^ cells were used for TEAD4 ChIP. Based on a size of 5 × 10^6^ cells, the cell pellets were lysed with 200 μl of cell lysis buffer (10 mM Tris-Cl pH 8.0, 10 mM NaCl, 0.2% NP-40) with protease inhibitor (Roche, 11697498001) and 1 mM DTT for 10 min on ice. The nuclei were isolated by centrifugation at 7,400 rpm for 30 seconds and gently resuspended in 200 μl of nuclear lysis buffer (50 mM Tris-Cl pH 8.0, 10 mM EDTA, 1% SDS) with protease inhibitor and 1 mM DTT. The lysates were sonicated for 10 cycles (30 seconds on/30 seconds off) and then incubated for 1 hour with 10 μg rabbit IgG and 10 μl of Protein A magnetic beads (Invitrogen, 10001) for pre-clearing. The immunoprecipitation was conducted with 10 μl of Protein A magnetic beads and 1 μg of the H3K27ac antibody (Abcam, ab4729) or 200 μl of TEAD4 antibody (Santa Cruz, sc-101184) overnight at 4°C on a rotator. The next day, the immunocomplexes were washed with IP Wash I Buffer, High salt buffer, IP Wash II buffer, and a final wash with TE buffer (pH 8.0). The washed immunocomplexes were eluted with 200 μl of elution buffer (1% SDS and 0.1 M NaHCO3) for 1 hour at 45°C on a thermomixer at 1,000 rpm. The eluates were de-crosslinked with the addition of 0.25 M NaCl and RNase A (1 μg/μl) and then incubated overnight in a 65°C water bath. The next day, after a 2-hr incubation with 4 μl of Proteinase K (NEB, P8107S), the ChIP DNA was purified with a QIAquick PCR purification kit (QIAGEN, 28106). 2 μl of 1:10 diluted ChIP DNA was subjected to ChIP-qPCR with 1 μl 10 μM primer and SYBR Green Master Mix. The negative region primer set is designed to amplify regions without ChIP-seq signals in each gene region. The following primers were used for ChIP-qPCR : hWWTR1 Negative region, 5’-GATGGTCAACTTTGGGGCAA-3’ (forward) and 5’-TGTTGCTTTCCACATTGCCA-3’ (reverse); hWWTR1 K27ac region, 5’-CCGCTCAGACCTGCATCT-3’ (forward) and 5’-TCAGGCCACTTTCCCTTTGA-3’ (reverse); hCD36 Negative region, 5’-TGAGTGAGAACATGCGGAGT-3’ (forward) and 5’-TGAAGCTGGAAACCGTCATTC-3’ (reverse); hCD36 K27ac region, 5’-CTGTCATTGGTGCTGTCCTG-3’ (forward) and 5’-TCTTCTGGATAAGCAGGTCTCC-3’ (reverse); hCD36 TEAD4 BS, 5’-CAACCCACATTCTGTTCGCA-3’ (forward) and 5’-TCTCTTCCCTTGTCTCAGCA-3’ (reverse).

### ChIP-seq library construction

ChIP-seq libraries were constructed using 40 μl of purified ChIP DNA and a NEXTflexTM ChIP-seq kit (PerkinElmer, NOVA-5143-02) according to the manufacturer's instructions. ChIP DNA was endrepaired and size-selected using AMPure XP beads (Beckman, A63881). Other procedures from adenylation to PCR amplification were done according to standard ChIP-seq library construction protocols. The quality of the resulting ChIP-seq libraries was determined using a Bioanalyzer and the High Sensitivity chip (Agilent). The average size of the ChIP-seq libraries ranged from 250 to 350 bp. For multiplexing, equimolar quantities of each library were combined while considering the sequencing depth per sample (20 to 40 million reads per library). The ChIP-seq libraries were sequenced using an Illumina NextSeq platform with single-end reads of 76 bases.

### Bioinformatic analyses of ChIP-seq

ChIP-seq analyses were performed as previously described (Roe et al., 2017). For alignment of ChIPseq reads, raw reads were mapped to the reference human (hg19) genome assemblies using Bowtie2, and duplicated reads were removed using SAMtools. The makeBigWig tool (in the HOMER suite) was used to generate bigWig files for visualization with the UCSC genome browser.

### Patient samples

A total of 81 CML samples (39 CP including NEL and CHR samples, 3 AP, and 39 BP) from bone marrow (BM) and peripheral blood (PB) were obtained from 49 CML patients. Among them, 8 patients had serial samples. Mononuclear cells (MCs) were isolated by Ficoll-Paque (GE Healthcare) density gradient centrifugation. Samples were frozen in 10% dimethyl sulfoxide (DMSO; Sigma-Aldrich) in fetal bovine serum (Sigma-Aldrich), stored in liquid nitrogen, and later thawed for analysis. All human samples were obtained from the Korea Leukemia Bank and the protocol was approved by the Institutional Review Board. Patient consent was obtained in accordance with the Declaration of Helsinki. For immunofluorescence, immunoblotting, qPCR, and cell viability assays of the patient cells, we thawed the cell stocks and centrifuged them at 800g for 5min to remove their supernatants. Every experiment was finished within 7 days to ensure proper cell viability. To verify recurrent FLT3 mutations, patient cDNA samples were amplified and sequenced with FLT3 primers (F, 5’- GCAATTTAGGTATGAAAGCCAGC - 3’; R, 5’- TTTTACAGGCAGACGGGCAT - 3’).

### Animal experiments

NOD/SCID mice were purchased from Koatech. For tumor xenograft models, K562-FLT3-IR cells (5 x 10^6^) in 1/3 matrigel (BD biosciences, 354248) were injected subcutaneously into 6-week-old male mice. Ten mice were assigned to each group. Four days after injection, imatinib (100 mg/kg), quizartinib (30 mg/kg), ponatinib (30 mg/kg), or vehicle (22% hydroxypropyl-β-cyclodextrin for quizartinib and 0.5% sodium carboxymethyl cellulose for imatinib and ponatinib) was administered 6 days per week by oral gavage. Tumor height and width were measured with calipers to calculate tumor volume (= width^2^ x height/2). Mice were sacrificed 10 weeks after the experiment began. All animal experiments were approved by the Yonsei University Institutional Animal Care and Use Committee (Documentation #201610-435-02).

### Statistics and reproducibility

All quantitative data were obtained from at least three independent biological replicates. Data are presented as means ± standard deviation (s.d.) unless otherwise noted in the figure legends. Statistical differences between two groups were examined using two-tailed, unpaired or paired Student's t-tests and one-way analyses of variance (ANOVA) with Bonferroni corrections for multiple comparisons. Statistical tests were performed using the GraphPad Prism 9.0 software (GraphPad Software, CA, USA). Two-sided p-values of less than 0.05 were considered significant.

## Results

### FLT3 expression promotes resistance to BCR::ABL1 TKIs in BP-CML cells

**Fig. 1:**
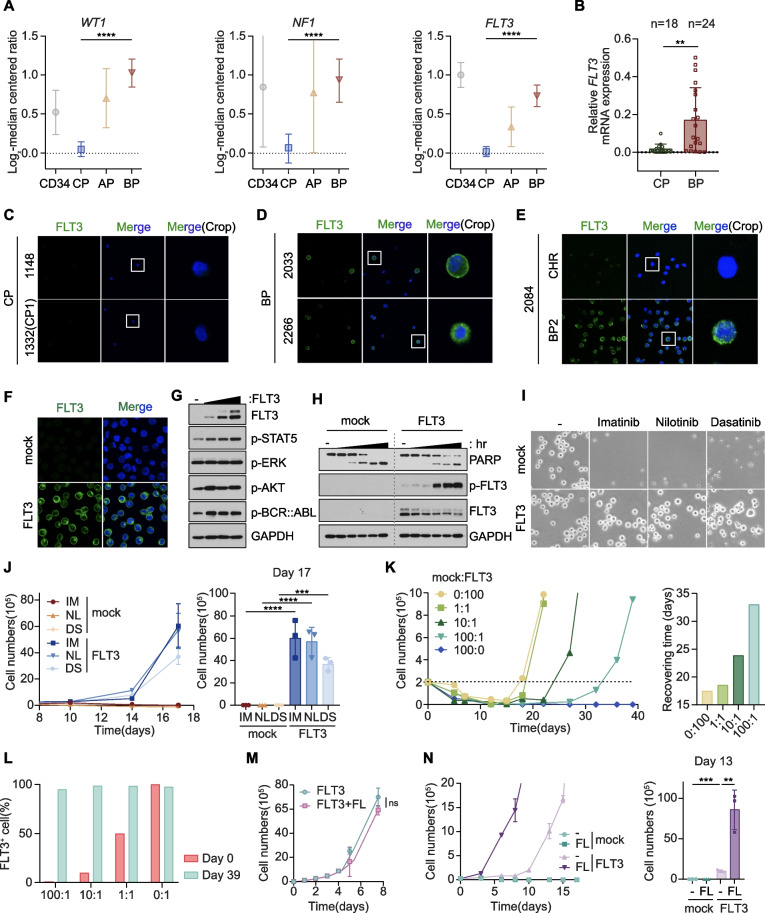
FLT3 activation in BP-CML cells promotes drug resistance to BCR::ABL1 TKI. **A** Analysis of the most highly increased AML driver genes in BP compared to CP-CML patients. The CML patients’ expression data were obtained from the NCBI Gene Expression Omnibus database (Accession no. GSE4170). Normal CD34 + cells (CD34), *n* = 7; CP, *n* = 57; AP, *n* = 9; BP, *n* = 33. p-values were calculated using one-way ANOVA with Bonferroni corrections for multiple comparisons. Error bars are means of  ± SD. ****p < 0.0001. **B** qPCR analysis of FLT3 mRNA levels in CP (*n* = 18) and BP (*n* = 24) CML patient cells. p-values were calculated using Student’s t-test and error bars are means of ± SD. **p < 0.01. **C-**E Immunofluorescence images of the cell surface localization of FLT3 (green) in BMMC samples derived from BP-CML patients 2033, 2266, and 2084 (BP2) compared to CP-CML samples 1148, 1332 (CP1), and 2084 (CHR). DAPI (blue) was used as a nuclear marker. CHR, complete hematological response. **F** Immunofluorescence images of the ectopic expression of FLT3 (green) on the cell surface in K562 cells. DAPI (blue) was used as a nuclear marker. **G** Immunoblotting of downstream signaling components induced by dose-dependent FLT3 expression in K562 cells. **H** Immunoblotting of imatinib-induced apoptosis in K562-mock and -FLT3 cells. PARP cleavage was measured in cells treated with 1 μM imatinib for 6, 18, 24, 28 and 72 h. **I** and **J** Cell images at day 17 (**I**) and growth curves (**J**) of control and K562-FLT3 cells treated with 1 μM imatinib, 20 nM nilotinib, and 1 μM dasatinib. **K** Growth curves of K562-FLT3 cells combined with control cells at different ratios (left). Recovery times for each combination after 1 μM imatinib treatment were recorded when cell numbers reached 2 × 10^5^ cells/well (right). **L** FACS analysis after dilution assay for the measurements of FLT3-positive cell percentages at day 0 and day 39. **M** Growth curves of K562-FLT3 cells with or without 20 ng/ml FLT3 ligand treatment. ns, not significant (*p* > 0.05). **N** Growth curves of control and K562-FLT3 cells treated with 1 μM imatinib with or without 20 ng/ml FLT3 ligand (left). Cell numbers were counted on day 13 (right). **p < 0.01, ***p < 0.001; ns, not significant (*p* > 0.05). **J**, **M–N** All p-values were calculated using Student’s t-test and error bars are means of triplicates ± SD. A p-value of less than 0.05 indicates a statistical difference

CML is unique because it arises from a single driver oncogene, BCR::ABL1 fusion tyrosine kinase, which has been targeted with successive generations of TKIs. Responses to TKI treatment, however, are short lived in advanced phase CML patients, which phenotypically resembles AML fueled by leukemic stem cells that are resistant to current treatment modalities [2]. Therefore, to test whether driver genes of AML might contribute to malignant reprogramming in BP-CML, we examined the expression levels of 27 AML driver genes [11] based on their distinct expression patterns in each phase of CML, according to data obtained from the NCBI Gene Expression Omnibus database (GSE4170). We found that BP-CML progression is associated with a significant induction of the AML driver genes WT1, NF1, and FLT3. Other AML drivers (e.g., EZH2, IDH2, KRAS, and MLL) remained unaffected or reduced during CML progression (Fig. 1A, Table S1).

Due to the advent of FLT3 targeted therapy for AML patients in 2017 with the approval of midostaurin [12], we aimed to uncover the pathophysiological role of FLT3 in BP progression and reevaluate the use of FLT3 inhibitors for CML patients. Remarkably, in our cohort of stage-specific CML patients, we confirmed significant upregulation of FLT3 transcripts in bone marrow mononuclear cells (BMMCs) isolated from BP-CML patients, whereas CP-CML samples showed hardly any FLT3 expression (Fig. 1B). The cell surface expression of FLT3 was apparent in BMMCs derived from BP-CML, but not from CP-CML patients (Fig. 1C and D). Consistently, paired serial samples showed strong cell surface expression of FLT3 in BP but not in CHR (complete hematological response) samples (Fig. 1E).

To recapitulate the emergence of FLT3 expression in BP-CML patients, we generated the K562-FLT3 cell line that stably expresses ectopic FLT3 protein in a CML cell line. We confirmed the expression and localization of FLT3 on the plasma membrane of K562-FLT3 cells (Fig. 1F). Dose dependent expression of FLT3 elicited minimal perturbation of p-BCR::ABL1 as well as p-STAT5, p-ERK, and p-AKT signaling, which are well-established downstream effectors of FLT3 in AML (Fig. 1G) [13]. In addition, FLT3 expression hardly affected cell cycle distribution or cell proliferation, suggesting that, apart from its role in AML, FLT3 does not endow CML cells with growth advantages (Fig. S1A and B). Next, because BP-CML patients acquire significant drug resistance, we asked whether FLT3 confers TKI resistance in CML cells. Time course treatment with imatinib triggered cell death in control cells shown by increased PARP cleavage. In K562-FLT3 cells, however, imatinib-induced FLT3 upregulation dramatically rescued cell viability (Fig. 1H and S1C). In addition to the acquisition of imatinib resistance, FLT3 activation further conferred resistance to the second generation TKIs, nilotinib and dasatinib, thus promoting unrestrained cell growth even in the presence of TKIs (Fig. 1I, J, and S1C). Next, we treated imatinib to individual clones generated from K562-FLT3 single cells to verify whether TKI-induced FLT3 activation was due to clonal selection or through an intrinsic mechanism. Interestingly, each clone showed FLT3 upregulation upon acquisition of TKI resistance, suggesting that inhibition of BCR::ABL1 by TKI could be eliciting FLT3 activation (Fig. S1D). Thus, we depleted BCR::ABL1 by siRNA to recapitulate TKI-induced BCR::ABL1 inhibition. Loss of BCR::ABL1 triggered FLT3 activation, which demonstrate that TKI-induced suppression of BCR::ABL1 directly or indirectly evokes FLT3 activation (Fig. S1E).

To determine whether a limited number of FLT3^+^ CML cells is sufficient to repopulate after TKI treatment, we diluted FLT3^+^ and FLT3^−^ cells in different ratios and then subjected them to imatinib treatment. Dilution analysis demonstrated that, although the lower FLT3^+^ ratios required longer period to repopulate, even those with extremely low numbers of FLT3^+^ CML cells were sufficient to eventually replace the whole population after acquiring imatinib resistance (Fig. 1K and L, S1F). FLT3 ligand (FL) activates FLT3 by triggering receptor dimerization and autophosphorylation [14]. Therefore, we next asked how ligand stimulation affects FLT3-mediated cell growth and drug responses. Consistent with the role of FLT3 in CML cells, FL treatment did not affect cell proliferation of either control or FLT3^+^ cells (Fig. 1M, S1G). FL stimulation did, however, enhance the acquisition of imatinib resistance of K562-FLT3 cells (Fig. 1N, S1H). These results demonstrate that the aberrant expression and activation of FLT3 is a critical determinant of BP progression and TKI resistance in BP-CML patients.

### Hippo transducers TAZ and TEAD mediate FLT3-induced drug resistance in BP-CML

**Fig. 2:**
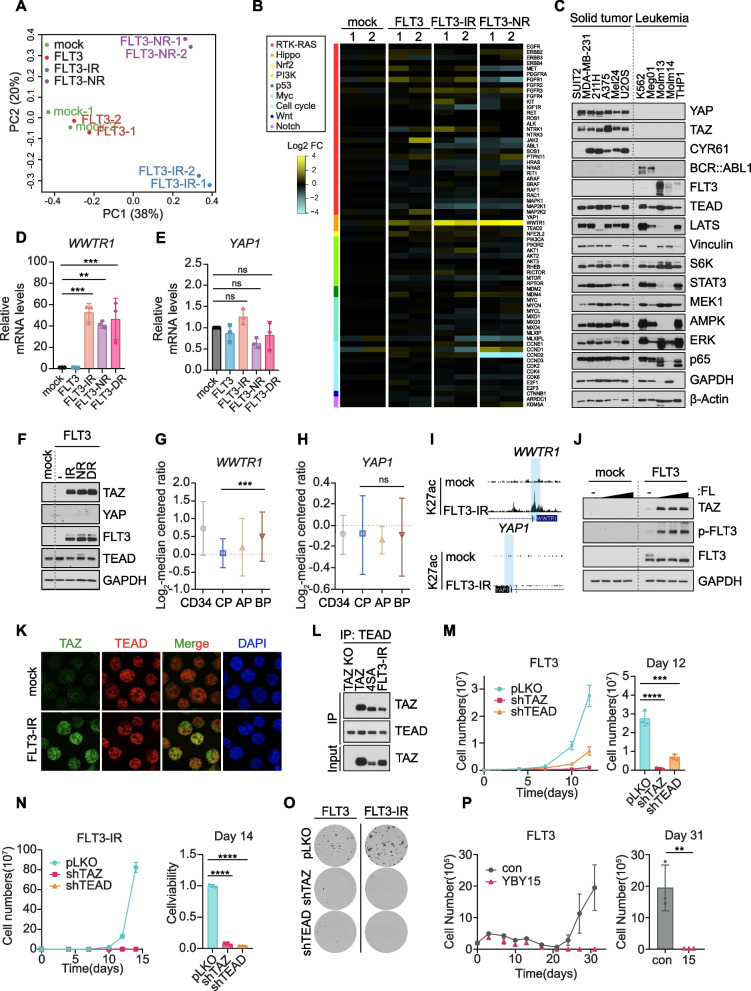
The Hippo transducers TAZ and TEAD mediate FLT3-induced drug resistance in BP-CML. **A** PCA analysis of the gene expression profiles of K562-mock, FLT3, FLT3-IR, and -NR cells. **B** Heatmap showing the relative expression of pro-tumorigenic factors within the major oncogenic pathways (color-coded as indicated in the legend) in RNA-seq data from K562-mock, K562-FLT3, K562-FLT3-IR, and NR cells. **C** Immunoblotting analysis comparing the expression patterns of various proteins in solid tumors and leukemia cell lines. **D** and **E** qPCR analyses of WWTR1 (**D**) and YAP1 (**E**) mRNA expression levels in K562-mock, FLT3, FLT3-IR, and FLT3-NR cells. **p < 0.01, ***p < 0.001; ns, not significant (*p* > 0.05). **F** Immunoblotting analysis of TAZ and YAP in K562-mock, FLT3, and TKI-resistant FLT3-IR, -NR, and -DR cells. **G** and **H** Expression of WWTR1 (**G**) and YAP1 (**H**) transcript levels in normal CD34 + cells (CD34) and each CML phase were analyzed from the NCBI Gene Expression Omnibus database (accession no. GSE4170). CD34, *n* = 7; CP, *n* = 57; AP, *n* = 9; BP, *n* = 33. *p*-values were calculated using Student’s t-test and error bars are means of ± SD. ***p < 0.001; ns, not significant (*p* > 0.05). **I** ChIP-seq analysis showing histone H3K27 acetylation (H3K27ac) profiles for the WWTR1 and YAP1 loci in K562-mock and -FLT3-IR cells. The promoter regions for each gene are highlighted. **J** Immunoblotting analysis of TAZ induction and FLT3 activation in K562-mock and K562-FLT3 cells treated with FLT3 ligand (0, 10, 30, and 100 ng/ml) for 36 h. **K** Immunofluorescence images of the nuclear localization TAZ (green) and TEAD (red) in K562-FLT3-IR cells. **L** Immunoprecipitation analysis identifying the protein–protein interactions between endogenous TAZ with TEAD in K562-FLT3-IR cells. Ectopic expression of flag-TAZ or flag-TAZ-4SA (a constitutively active TAZ mutant) were used as positive controls. TAZ knockout (KO) cells were used as a negative control. **M** Acquisition of TKI resistance in K562-FLT3 cells transduced with shRNAs targeting TAZ or TEAD. Growth curve of cells treated with 1 μM imatinib (left) and a bar graph showing the indicated cell numbers measured on day 12 (right). ***p < 0.001, ****p < 0.0001. **N** Restoration of TKI sensitivity in K562-FLT3-IR cells transduced with shRNAs targeting TAZ or TEAD. Growth curve analysis of cells treated with 1 μM imatinib (left) and a bar graph showing the indicated cell numbers measured on day 14 (right). ****p < 0.0001. **O** Colony formation assay in K562-FLT3 cells treated with 3 μM imatinib and 20 ng/ml FL (left column) and K562-FLT3-IR cells treated with 3 μM imatinib for 3 weeks (right column) after knockdown of TAZ or TEAD. **P** Growth curves (left) and viability (right) of K562-FLT3 cells treated with 1 μM imatinib with or without TEAD inhibitor YBY-15 (30 μM). ****p < 0.0001. **D-E**, **M–N**, **P** All p-values were calculated using one-way ANOVA with Bonferroni corrections for multiple comparisons (**D**-**E**, **M**–**N**) and Student’s t-test (**P**). Error bars are means of triplicates ± SD. A *p*-value of less than 0.05 indicates a statistical difference

To identify the downstream effectors of FLT3 in drug resistant CML cells, we performed RNA-seq analysis of FLT3-dependent TKI-resistant K562-FLT3-IR (imatinib-resistant), K562-FLT3-NR (nilotinib-resistant), and FLT3-independent K562-IMR (spontaneous imatinib-resistant), K562-NLR (spontaneous nilotinib-resistant) [15] cells and examined key oncogenes and signaling pathways frequently altered in cancers. Transcriptome analyses indicated that FLT3-mediated TKI resistance significantly alters the expression profiles of CML cells (Fig. 2A, S2A). Surprisingly, among the many pro-tumorigenic factors, we found that TKI-resistant K562-FLT3-IR and -NR cells, but not FLT3-independent IMR and NLR cells, expressed significantly higher levels of TAZ (encoded by the *WWTR1* gene) than control cells or TKI-untreated K562-FLT3 cells (Fig. 2B and S2B). YAP and TAZ are transcriptional coactivators of the Hippo signaling pathway that bind TEAD family transcription factors and play critical roles in tumorigenesis and regeneration [16, 17]. Notably, unlike solid tumor cells, YAP and TAZ are hardly expressed in the hematopoietic lineages (Fig. 2C, S2C) and are dispensable for physiologic and malignant hematopoiesis [18, 19]. Thus, we were surprised to find aberrant expression of TAZ, but not YAP, in CML cells resistant to both first and second generation TKIs in vitro (Fig. 2D-F), as well as in BP-CML patients compared to those in CP or AP (Fig. 2G and H). Compared to control or IMR cells, we found that FLT3-mediated drug resistance evoked histone H3K27ac accumulation specifically at the promotor region of *WWTR1*, but not at that of *YAP*, indicating epigenetic alteration accounts for TAZ induction in TKI-resistant K562-FLT3-IR cells (Fig. 2I and S2D). Moreover, FL stimulation potentiated TAZ expression (Fig. 2J), which was consistent with our finding that FL augments TKI resistance in FLT3-expressing cells (Fig. 1N).

Consistent with the role of TAZ-TEAD axis in solid tumors, we next asked whether TEAD mediates the downstream function of FLT3-TAZ signaling, which has not been explored in the pathogenesis of CML. Similar to the expression of ectopic TAZ-WT and constitutively active TAZ-4SA, we observed both nuclear and cytoplasmic localization of endogenous TAZ in TKI-resistant K562-FLT3-IR and -NR cells (Fig. 2K, S2E). We also verified the protein–protein interaction between TAZ and TEAD (Fig. 2L). To determine whether the TAZ-TEAD axis is important in both the acquisition as well as the maintenance of FLT3-mediated TKI resistance, we depleted TAZ or TEAD in K562-FLT3 and FLT3-IR cells (Fig. S2F and G). Cells were subsequently treated with each TKI for 2 weeks. The absence of TAZ or TEAD markedly suppressed cell proliferation and colony formation by blocking FL-stimulated K562-FLT3 cells from acquiring TKI resistance, and by restoring drug sensitivity to TKI-resistant K562-FLT3 cells (Fig. 2M-O, S2H-L). Consistent with these results, we also found that a small molecule TEAD inhibitor YBY-15 [20] suppressed the acquisition of imatinib resistance in K562-FLT3 cells (Fig. 2P). Here, we establish Hippo transducer TAZ as a critical downstream effector of FLT3 signaling in CML. Our results show that FLT3 activation triggers the epigenetic activation of TAZ in BP-CML cells, which subsequently interacts with TEAD to elicit TKI resistance.

### FLT3-JAK-STAT3 and Hippo-mediated TAZ regulation leads to TKI resistance in BP-CML

**Fig. 3:**
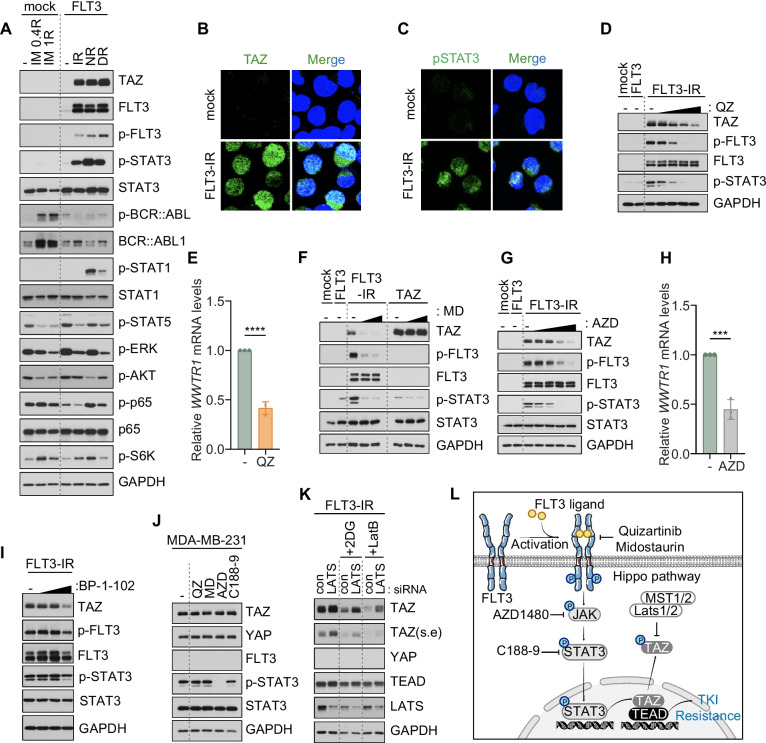
Acquisition of TKI resistance via FLT3-JAK-STAT3-TAZ and Hippo-TAZ signaling in BP-CML. **A** Immunoblotting analysis of FLT3, BCR::ABL1, and their downstream signaling components in spontaneous imatinib-resistant K562 cells (IM-0.4R, 0.4 μM imatinib-resistant cells; IM-1R, 1 μM imatinib-resistant cells) compared to FLT3-mediated TKI-resistant K562-FLT3-IR, -NR, and -DR cells. **B** and **C** Representative immunofluorescence images of the nuclear localization of TAZ (green) and p-STAT3 (green) in K562-FLT3-IR cells. **D** Immunoblotting analysis of FLT3-pSTAT3-TAZ signaling components in K562-FLT3-IR cells treated for 16 h with increasing doses of quizartinib (QZ; 0, 3, 10, 30, 100 nM). **E** Measurements of TAZ transcript levels in K562-FLT3-IR cells treated for 16 h with 30 nM quizartinib. ****p < 0.0001. **F** Immunoblotting analysis of FLT3-pSTAT3-TAZ signaling components in K562-FLT3-IR and K562-TAZ cells treated with various doses of midostaurin (MD; 0, 0.1, 0.3 μM) for 16 h. **G** Immunoblotting analysis of FLT3-pSTAT3-TAZ signaling components in K562-FLT3-IR cells treated with various doses of JAK inhibitor AZD-1480 treatment (AZD; 0, 0.1, 0.3, 1, 3 μM) for 16 h. ***p < 0.001. **H** Measurements of TAZ transcript levels in K562-FLT3-IR cells treated for 16 h with 2.5 μM AZD-1480. **I** Immunoblotting analysis of FLT3-pSTAT3-TAZ signaling components in K562-FLT3-IR cells treated with various doses of BP-1–102 (0, 1, 5, 10 μM) for 16 h. **J** Immunoblotting analysis of FLT3-pSTAT3-TAZ signaling components in MDA-MD-231 breast cancer cells after treatments with 30 nM quizartinib (QZ), 0.3 μM midostaurin (MD), 3 μM AZD-1480 (AZD), and 30 μM C188-9. **K** Immunoblotting analysis of TAZ protein in control or LATS1/2-depleted K562-FLT3-IR cells treated with the Hippo pathway activators 2-DG (6.25 mM) or LatB (1 μg/ml) for 16 h. s.e, short exposure. **L** Schematic illustrating the transcriptional and post-translational mechanisms that regulate TAZ in BP-CML. **E**, **H** All p-values were calculated using Student’s t-tests and error bars are means of triplicates ± SD. A *p*-value of less than 0.05 indicates a statistical difference

Next, we investigated the downstream components of FLT3 that promotes TAZ expression in CML cells. Notably, induction of TAZ specifically occurred in FLT3^+^ TKI-resistant cells, but not in spontaneous imatinib-resistant cell line, demonstrating the specific association between FLT3 and TAZ (Fig. 3A). We tested multiple FLT3 signaling components, including effectors of the STAT, MAPK, NFkB, Src, and PI3K pathways, as well as BCR::ABL1 signaling. Interestingly, the activation of transcription factor STAT3 showed the strongest correlation with TAZ induction in K562-FLT3-IR, -NR, and -DR cells (Fig. 3A). In FLT3-IR cells, both TAZ and p-STAT3 were localized in the nucleus (Fig. 3B and C). We tested inhibitors for various components to clarify the signaling pathway as well as molecular targets involved in TKI resistance. Since FLT3 is a major driver of AML, several classes of FLT3 inhibitors (e.g., midostaurin, gilteritinib, quizartinib) have been developed for the treatment of AML patients [10]. Thus, we tested whether FLT3 inhibitors impair FLT3 signaling and abolish TAZ expression in TKI-resistant CML cells. The FLT3 inhibitors quizartinib and midostaurin effectively suppressed phosphorylation of FLT3 and STAT3 in K562-FLT3-IR cells and blocked TAZ mRNA and protein expression (Fig. 3D-F). These results indicate TAZ as a STAT3 target gene in CML cells. Midostaurin failed to suppress the ectopically expressed TAZ protein, indicating that FLT3 regulates TAZ mRNA expression, but does not affect TAZ protein stability (Fig. 3F). Next, we found that inhibiting JAK or STAT3 blocked the induction of TAZ in FLT3-IR cells (Fig. 3G-I). This indicates that FLT3-JAK-STAT3 signaling mediates TAZ expression in TKI-resistant CML cells. We conducted further investigations to determine whether FLT3-TAZ signaling is common in leukemia cells. Another CML cell line, AR230, recapitulated FLT3 signaling via acquisition of TKI resistance (Fig. S3A). In contrast, AML cells that express endogenous FLT3, however, hardly showed TAZ induction even upon imatinib treatment, which suggests FLT3-TAZ signaling may be a unique mechanism of CML (Fig. S3B). We further asked whether FLT3-JAK-STAT3 signaling induces TAZ expression similarly in solid tumor cells. Treatment with each inhibitor in breast and gastric cancer cells had negligible effects on TAZ expression, suggesting that the FLT3-JAK-STAT3-TAZ-TEAD signaling is unique to FLT3^+^ CML cells (Fig. 3J, S3C). Unlike YAP and TAZ, hematopoietic malignancies express Hippo pathway components, such as the LATS1/2 kinases, that could control YAP/TAZ protein stability and nucleocytoplasmic localization upon their induction [16]. Thus, we asked whether Hippo pathway subsequently governs TAZ protein stability in BP-CML. We treated control and LATS1/2-depleted K562-FLT3-IR cells with the well-defined Hippo pathway-activating stressors 2-DG or LatB, which activate LATS1/2 by suppressing glycolysis or actin polymerization, respectively [21, 22]. Both 2-DG and LatB induced TAZ degradation in FLT3-IR cells that were significantly blocked by LATS depletion (Fig. 3K). These results demonstrate that TAZ activity can be regulated by the Hippo pathway in TKI-resistant CML cells. Our results establish 1) FLT3-JAK-STAT3 axis as the transcriptional module of TAZ, and 2) Hippo pathway as the post-translational module of TAZ. Together, these pathways synergistically promote FLT3-mediated drug resistance in BP-CML (Fig. 3L).

### Activation of FLT3-TAZ signaling correlates with less favorable prognosis in BP-CML

**Fig. 4:**
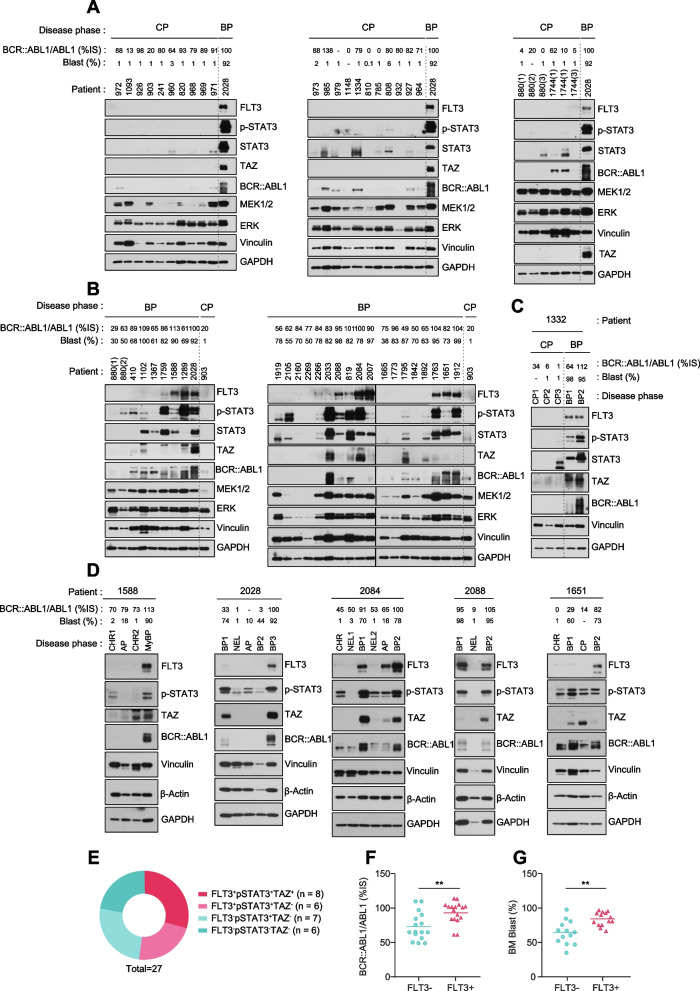
Activation of FLT3-TAZ signaling correlates with less favorable prognosis in BP-CML. **A** Immunoblotting analysis of FLT3-pSTAT3-TAZ components and BCR::ABL1 in 27 clinical samples derived from CP-CML patients. BP-CML patient sample 2028 was used as a control for comparing protein expression levels between CP and BP patients. CML prognostic factors such as disease phase, BCR::ABL1/ABL1 (%IS), and blast % are indicated above each patient specimen. **B** Immunoblotting analysis of 27 clinical samples derived from BP-CML patients. CP-CML patient sample 903 was used as a control for comparing protein expression levels between CP and BP patients. **C** and **D** Immunoblotting analysis of paired serial samples from individual patients. CHR, complete hematologic response; MyBC, myeloid blast crisis; NEL, no evidence of leukemia. **E** Pie chart showing the co-expression patterns between FLT3, pSTAT3, and TAZ proteins in BP-CML patients (*n* = 27). **F** and **G** Clinical relevance of FLT3 expression level and prognostic factors for CML, including BCR::ABL1/ABL1 ratio (IS%) (F) and blast percentage (**G**), in BP-CML patients. FLT3^−^, n = 13; FLT3.^+^, n = 14. All p-values were calculated using Student’s t-test and error bars are means ± SD. A *p*-value of less than 0.05 indicates a statistical difference. **p < 0.01

As FLT3 and TAZ transcripts were consistently upregulated in BP-CML patients (Figs. 1A, 2G), we decided to further investigate the clinical relevance of FLT3-TAZ signaling from our cohort, which consists of CP and BP-CML specimens as well as paired CP-to-BP serial samples. Consistent with the transcript level, we found very low expression levels of FLT3 signaling components in the CP samples. We were surprised, however, to observe dramatic increase in FLT3 protein levels in approximately half of the BP-CML patients (14 out of 27 BC patients) from our cohort (Fig. 4A-C). The expression of FLT3 in BP-CML samples were comparable to those in K562-FLT3 and AML cells (Fig. S4A and B). In addition, p-STAT3 and TAZ, which were identified to act downstream of FLT3, were expressed at significantly higher levels in BP-CML patients, which correlated to a large extent with FLT3 and the prognostic factors of CML (Fig. S4C). Next, we investigated the correlation between BP progression and FLT3-TAZ axis activation by examining the CP-to-BP paired serial samples. We found dramatic loss of FLT3 expression in samples from patients who achieved either CP, complete hematologic response (CHR), or no evidence of leukemia (NEL) after TKI treatment. Massive accumulation of FLT3^+^ BMMCs was evident, however, in AP and BP samples, as well as in the second and third BP following TKI failure within the paired serial samples (Fig. 4C and D). Overall, these results confirm the activation of FLT3 signaling in a considerable portion of BP patients, including subsets who co-expressed either pSTAT3 or TAZ (Fig. 4E).

To verify the prognostic significance of FLT3 signaling in BP progression, we compared critical prognostic factors of CML, including blast percentage, BCR::ABL1/ABL1 (%IS) ratio, BCR::ABL1 mutations, and overall patient survival between FLT3^−^ and FLT3^+^ subgroups in BP-CML patients. Intriguingly, prognostic factors of CML such as blast percentage (86% vs. 69%) and BCR::ABL1 /ABL1^IS^ ratio (97.6% vs. 65.4%) were significantly higher in FLT3^+^ BP patients than FLT3^−^ BP patients. Moreover, the FLT3^+^ BP-CML subgroup showed significantly shorter times from 1) diagnosis to death (26.8 vs 206 months) and from 2) BP progression to death (11.7 vs 66 months) than the FLT3^−^ BP-CML subgroup. These data suggest that FLT3^+^ patients manifest more aggressive CML pathologies and show worse overall survival compared to FLT3^−^ BP patients (Fig. 4F and G, S4D**,** Table S2). Next, we asked whether BCR::ABL1 driver mutations associated with TKI-resistance and BP progression correlated with FLT3 expression in BP-CML patients. Both FLT3^+^ and FLT3^−^ patients showed similar incidence of recurrent BCR::ABL1 mutations, suggesting that FLT3 induction occurs independent of BCR::ABL1 mutation status (Table S3). To confirm that FLT3 induction was irrelevant to BCR::ABL1 mutations, we analyzed Ba/F3 cells expressing either BCR::ABL1 wild type (WT) or BCR::ABL1 harboring single or compound kinase domain mutations. In line with patient results, recurrent BCR::ABL1 mutations failed to elicit FLT3 and TAZ expression (Fig. S4E-G). Moreover, recurrent BCR::ABL1 mutations were not found in TKI-resistant K562-FLT3-IR, -NR, and -DR cells, and depletion of BCR::ABL1 did not affect FLT3-TAZ signaling (Fig. S4H and I). Together, these results indicate that activation of FLT3-TAZ signaling correlates with less favorable prognosis in BP-CML, which were independent of recurrent BCR::ABL1 mutations, thus demonstrate the prognostic impact of FLT3-TAZ axis in assessing CML progression.

### FLT3-TAZ axis promotes TKI resistance via CD36-mediated fatty acid uptake

**Fig. 5:**
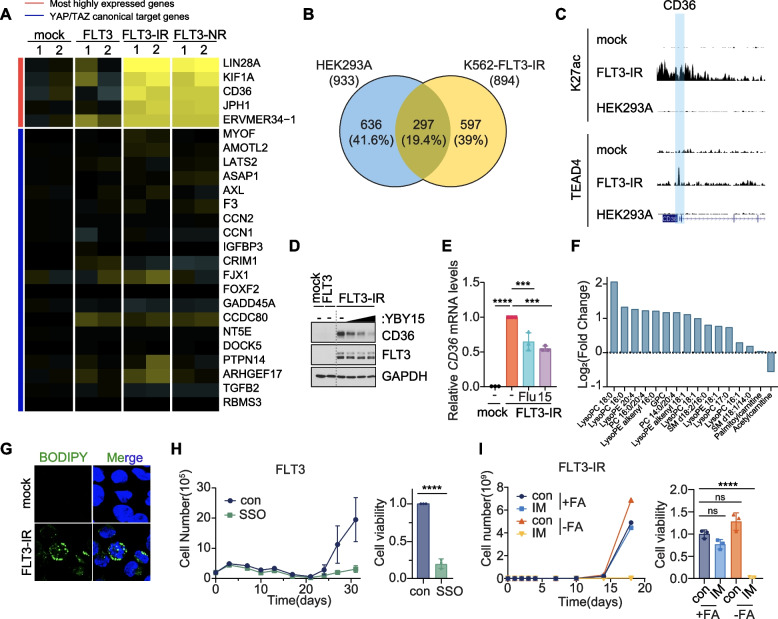
The FLT3-TAZ signaling promotes TKI resistance via CD36-mediated fatty acid uptake. **A** Heatmap analysis of RNA-seq data from K562-mock, -FLT3, and TKI-resistant K562-FLT3-IR and -NR. The most strongly enhanced genes are labeled with red and the canonical YAP/TAZ target genes are labeled with blue. **B** Venn Diagram of TEAD4 ChIP-seq results comparing the overlap of direct TEAD4 target genes in HEK293A and K562-FLT3-IR cells. **C** ChIP-seq analysis showing TEAD4 and H3K27ac enrichment at the CD36 promoter region (highlighted) in HEK293A, K562-mock, and K562-FLT3-IR cells. **D** Immunoblotting analysis of CD36 protein levels in K562-FLT3-IR cells after treatment with TEAD inhibitor YBY-15 (0, 5, 10, 20 μM) for 16 h. **E** Measurements of CD36 transcript levels in K562-FLT3-IR cells treated with TEAD inhibitors flufenamic acid (Flu; 200 μM) or YBY-15 (15; 30 μM). **F** Metabolomic analysis of intracellular fatty acid derivatives in K562-FLT3-IR cells compared to K562-mock cells. **G** Lipid droplet analysis using BODIPY (green) staining in K562-mock and K562-FLT3-IR cells. DAPI (blue) was used as a nucleus marker. **H** Measurement of cell growth (left) and cell viability (right) in K562-FLT3 cells treated with imatinib (1 μM) treatment with or without CD36 inhibitor, SSO (200 μM). ****p < 0.0001. **I** Measurement of cell growth (left) and cell viability (right) of K562-FLT3-IR cells treated with normal or fatty acid-free medium with or without imatinib (1 μM). ****p < 0.0001; ns, not significant. **E**, **H-I**, All p-values were calculated using one-way ANOVA with Bonferroni corrections for multiple comparisons (**E**, **I**) and Student’s t-test (**H**). Error bars are means of triplicates ± SD. A p-value of less than 0.05 indicates a statistical difference

To identify the molecular target of TEAD that confers FLT3-induced TKI resistance, we performed RNA-seq analysis in K562-FLT3-IR and -NR cells. Because the TAZ promotor is epigenetically silenced in CML cells prior to the acquisition of drug resistance (Fig. 2I), we hypothesized that FLT3-induced TAZ-TEAD may deliver a distinct transcriptional output in hematologic malignancies compared to that of adherent cells, which constitutively express TAZ protein (Fig. S2B). While the expression of canonical YAP/TAZ-TEAD target genes were mostly unaffected in K562-FLT3-IR and -NR cells, we found several genes highly increased in TKI-resistant FLT3 expressing cells, such as LIN28A, KIF1A, CD36, and JPH11, which were not previously known as TEAD target genes (Fig. 5A) [23]. These genes, however, were not highly ranked in FLT3-independent IMR or NLR cells (Fig. S5A). Consistent with these results, ChIP-seq analysis indicated that TEAD occupied a significant number of promoters (597 genes, 39%) that were unique to TKI-resistant CML cells (Fig. 5B). Among the highly altered genes in RNA-seq analysis (Fig. 5A), ChIP-seq results confirmed the direct binding of TEAD and enrichment of K27ac at the promoter region of CD36 only in K562-FLT3-IR cells, but not in K562-mock, -IMR, or HEK293A cells (Fig. 5C, S5B and C). In contrast, TEAD and K27ac accumulation at the promoter region of CTGF, a canonical TEAD target gene, was only apparent in HEK293A cells (Fig. S5D). We confirmed that the protein and mRNA levels of CD36 were significantly increased in K562-FLT3-IR cells, which were then abolished by the administration of TEAD inhibitors flufenamic acid or YBY-15 (Fig. 5D and E). These results establish the CD36 fatty acid transporter as a direct TEAD target gene in FLT3^+^ CML cells.

It is interesting to note that CD36-expressing leukemic stem cells (LSCs) in BP-CML have been shown to confer protective effect from anticancer therapies by the accumulation of lipid droplets [24, 25]. Among various fatty acid transporters, we found that CD36 was selectively upregulated in FLT3-IR and -NR cells (Fig. S5E). Global metabolomic analysis indicated that FLT3-IR cells showed significantly enhanced lipid contents, especially lysophosphatidylcholine and lysophosphatidylethanolamine (Fig. 5F) and cells treated with boron-dipyrromethene dye (BODIPY) showed stronger lipid droplet staining (Fig. 5G). To determine whether CD36-mediated fatty acid uptake is a critical determinant in FLT3-TAZ axis-mediated TKI resistance, we measured the viability of K562-FLT3 cells after treatment with TKI and a CD36 inhibitor. While K562-FLT3 cells acquire drug resistance within 3 weeks of imatinib treatment, co-administration of imatinib with a CD36 inhibitor dramatically prevented the acquisition of FLT3-induced drug resistance (Fig. 5H). Furthermore, we found that FLT3-IR, -NR, and -DR cells cultured in fatty acid-depleted medium restored TKI sensitivity (Fig. 5I, S5F and G). These results reveal CD36-mediated fatty acid uptake as critical downstream effector of TEAD and establish the FLT3-JAK-STAT3-TAZ-TEAD-CD36 axis as a novel signaling pathway that confers TKI resistance in BP-CML cells.

### Combined inhibition of BCR::ABL1 and FLT3 suppresses BP-CML tumor growth

**Fig. 6:**
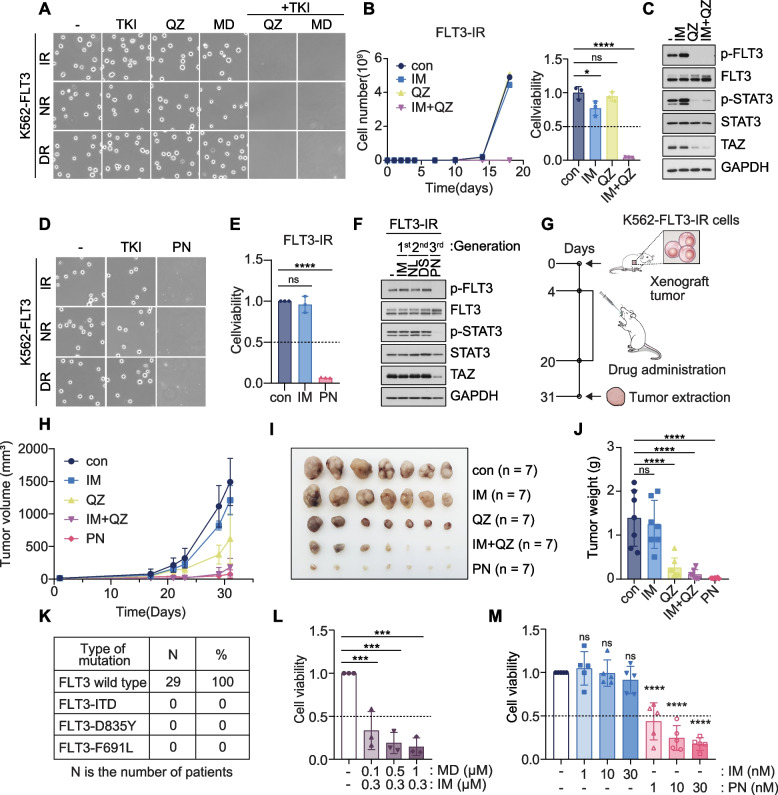
Combined inhibition of BCR::ABL1 and FLT3 suppresses BP-CML tumor growth. **A** Cell images of TKI-resistant K562-FLT3-IR, -NR, and -DR cells treated with either BCR::ABL1 TKI (1 μM imatinib, 20 nM nilotinib, 1 μM dasatinib), 30 nM quizartinib (QZ), 0.1 μM midostaurin (MD) or the combination of each BCR::ABL1 TKI with either quizartinib or midostaurin for 7 days. **B** Measurements of cell growth (left) and cell viability (day 18) (right) of K562-FLT3-IR cells subjected to single treatments with vehicle (con), 1 μM imatinib (IM), 30 nM quizartinib (QZ), or the combination of imatinib and quizartinib (IM + QZ). *n* = 3 per group. *p < 0.05, ****p < 0.0001; ns, not significant. **C,** Immunoblotting analysis of FLT3-pSTAT-TAZ signaling components in K562-FLT3-IR cells subjected to single treatments with 1 μM imatinib (IM), 30 nM quizartinib (QZ), or the combination of imatinib and quizartinib (IM + QZ) for 16 h. **D** Cell images of TKI-resistant K562-FLT3-IR, -NR, and -DR cells treated with the indicated BCR::ABL1 inhibitors as in (**A**) or with 10 nM ponatinib (PN). **E** Cell viability measurement for K562-FLT3-IR cells treated with 1 μM imatinib (IM) or 10 nM ponatinib (PN) treatment. ****p < 0.0001; ns, not significant (*p* > 0.05). **F** Immunoblotting analysis of FLT3-pSTAT-TAZ signaling components in K562-FLT3-IR cells treated with 1 μM imatinib (IM), 20 nM nilotinib (NL), 1 μM dasatinib (DS), or 10 nM ponatinib (PN) for 16 h. **G** Experimental procedure to analyze the therapeutic efficacy of combined treatment of imatinib and quizartinib (IM + QZ) or single treatment of ponatinib (PN) in K562-FLT3-IR xenograft model. **H** Tumor growth analysis of K562-FLT3-IR-derived xenografts in NOD/SCID mice treated with either vehicle (con), 100 mg/kg imatinib (IM), 30 mg/kg quizartinib (QZ), 15 mg/kg ponatinib (PN), or the combination of imatinib and quizartinib (IM + QZ). All drugs were delivered by daily oral gavage. n = 3 per group. **I** and **J** Tumor images (I) and tumor weights (J) of excised K562-FLT3-IR-derived tumors from each group (*n* = 7). ****p < 0.0001; ns, not significant. **K** Analysis of FLT3 driver mutations by cDNA sequencing of BP-CML patient samples. **L** Cell viability measurement of FLT3^+^ imatinib-resistant BP-CML patient-derived BMMCs subjected to combined treatment with imatinib (IM) and midostaurin (MD) for 5 days. *n* = 3 per group. ***p < 0.001. Independent patient information and results are shown in Fig. S5I. **M** Cell viability measurement of FLT3^+^ imatinib-resistant BP-CML patient-derived BMMCs subjected to treatment with either imatinib (IM) or ponatinib (PN) for 5 days. *n* = 5 per group. ****p < 0.0001; ns, not significant. Independent patient information and results are shown in Fig. S5J. **B**, **E**, **J**, **L-M** All p-values were calculated using one-way ANOVA with Bonferroni corrections for multiple comparisons and error bars are means ± SD. A *p*-value of less than 0.05 indicates a statistical difference

Targeted therapies of FLT3 have proven effective in the treatment in AML patients [10]. Based on our findings, we asked whether the FLT3 inhibitors, which are developed for AML patients, could be repurposed for FLT3^+^ BP-CML patients to restore BCR::ABL1 TKI sensitivity and prevent BP progression. We found that single treatment with each corresponding BCR::ABL1 inhibitors had negligible effect on the growth of TKI-resistant K562-FLT3-IR, -NR, and -DR cells, whereas quizartinib partially suppressed cell growth in K562-FLT3-NR and -DR cells. Remarkably, co-administration of TKI with FLT3 inhibitors, however, promoted significant cell death of TKI-resistant K562-FLT3 cells, but not in IMR cells (Fig. 6A and B, S6A-C). We then depleted BCR::ABL1 in K562-IMR and -FLT3-IR cells to see if it affects FLT3-dependent TKI resistance. Upon imatinib treatment, BCR::ABL1-depleted control and IMR cells showed dramatic cell death, whereas FLT3-IR cell were unaffected, suggesting BCR-ABL1 is dispensable for FLT3-induced TKI resistance (Fig. S6D-F). Consistently, TKI-resistant FLT3-IR cells subjected to quizartinib treatment, either alone or in combination with BCR::ABL1 inhibitors, showed marked suppression of FLT3-JAK-STAT3-TAZ signaling (Fig. 6C, S6G and H). It is important to note that ponatinib, a third generation BCR::ABL1 targeted therapy, can potently inhibit FLT3 receptor phosphorylation and cell proliferation in AML cells with an IC_50_ value comparable to that required for BCR::ABL1 inhibition [26]. We were excited to find that, although single treatments with imatinib, nilotinib, or dasatinib, did not suppress cell growth, treatment with ponatinib alone recapitulated the efficacy of combined treatment of TKI with FLT3 inhibitor triggering cell death in all TKI-resistant K562-FLT3 cells (Fig. 6D and E, S6I and J). Moreover, in contrast to older generation TKIs, we found that ponatinib alone potently suppressed FLT3-TAZ signaling in all TKI-resistant K562-FLT3 cells (Fig. 6F, S6K). These data demonstrate that targeting the FLT3-TAZ axis with either the combination of FLT3 inhibitor with TKI or single treatment with ponatinib alone can abolish drug resistance and render CML cells vulnerable to continued TKI treatment.

Based on these results, we next asked whether dual inhibition of BCR::ABL1 and FLT3 could overcome TKI resistance in xenograft mouse models and eradicate patient-derived BP-CML cell growth. After injecting TKI-resistant FLT3-IR cells subcutaneously into NOD-SCID mice, drugs were orally administrated to each group accordingly (Fig. 6G). Administration of imatinib alone exerted negligible effect on the tumor growth and volume derived from FLT3-IR cells. We found that administration of quizartinib alone achieved significant therapeutic response in vivo. Moreover, the dual inhibition of BCR::ABL1 and FLT3 via combined treatment of imatinib with quizartinib further impaired tumor growth. Remarkably, single treatment with ponatinib significantly abolished tumor growth and recapitulated the therapeutic efficacy of combined therapy (Fig. 6H-J).

Finally, we asked how the inclusion of FLT3 inhibitor affects the viability of BMMCs isolated from FLT3^+^ BC-CML patients. Because FLT3 overexpression or FLT3 mutations, including FLT3-ITD (internal tandem duplication) and TKD (tyrosine kinase domain) mutations, are well-established AML drivers [10], we verified the mutational status of FLT3 in our patient cohort. Thus, we performed sequencing of specific regions of the FLT3 gene that harbor recurrent driver mutations. To our surprise, all BP-CML patients showed wild-type FLT3 sequences, and none of the recurrent FLT3 mutations were detected in our cohort (Fig. 6K, S6L). Therefore, to develop a treatment protocol that efficiently inhibits wild-type FLT3 for CML patients, we treated imatinib together with the first FDA-approved FLT3 inhibitor midostaurin. We found that midostaurin treatment restored TKI effectiveness and induced significant dose-dependent cytotoxicity in BMMCs isolated from imatinib-resistant BP-CML patients (Fig. 6L, S6M). Consistently, single treatment of ponatinib produced dose-dependent cytotoxicity, which was equivalent to that of the combined therapy (Fig. 6M, S6N). Our findings support combining FLT3 inhibitors with BCR::ABL1 TKIs or single treatment of ponatinib for the treatment of FLT3^+^ CML patients, which abolishes FLT3-TAZ signaling, restores TKI sensitivity, and enhances therapeutic efficacy.

## Discussion

In the present study, we explored the role of FLT3 in the pathogenesis of advanced phase CML. Our results propose unprecedented therapeutic strategies for the treatment of CML aimed at overcoming and preventing drug resistance and progression to the current frontline BCR::ABL1 targeted therapies. The precise mechanisms by which CML patients progress from the CP to BP remain unclear, and treatment of BP-CML continues to be a critical area of challenge [4, 6, 27]. Here, we report FLT3 as a therapeutic target of CML that activates FLT3-JAK/STAT3-TAZ-TEAD-CD36 pathway during BP progression and promotes the acquisition of BCR::ABL1 TKI resistance in BP-CML patients.

Treatment outcomes for BP-CML patients have suffered due to the lack of validated and reliable markers to predict blast crisis [3, 28]. Our study establishes FLT3 as a clinical and molecular prognostic marker for CML progression and acquisition of TKI resistance. Several studies have suggested the potential role of FLT3 in CML [29–31]. To our knowledge, our study provides the first comprehensive evidence and clinical relevance for such a role using clinical samples from a cohort of unpaired and paired CP-to-BP serial samples. Here, we verified the molecular mechanisms of FLT3-driven drug resistance, optimized methods for the detection of FLT3 protein expression and cellular localization, the correlation between FLT3 and CML prognostic factors, and the therapeutic strategies to tackle FLT3^+^ CML patients. We also further demonstrated the clinical feasibility of detecting FLT3 expression in CML specimens via immunoblotting, immunofluorescence, or PCR methods, as well as targeting FLT3^+^ CML cells with approved FLT3 inhibitors. Thus, classifying a new prognostic CML subgroup using FLT3 expression as a biomarker will facilitate the prediction of sudden onset of BP, provide guidelines for the rational management of advanced phase CML patients, and the selection of patients for targeted clinical trials.

Based on these results, our study suggests that co-administration of FLT3 inhibitors with BCR::ABL1 targeted therapies can overcome TKI resistance and BP progression in FLT3^+^ CML patients. Inclusion of FLT3 inhibitors, such as midostaurin and quizartinib, restored the cytotoxicity of first and second generation BCR::ABL1 TKIs, including imatinib, dasatinib, and nilotinib, thereby suppressing the emergence of highly resistant BP cells. It is important to note that recurrent mutations in FLT3, such as FLT3-ITD and FLT3-TKD, evoke drug resistance and limit the usage of certain FLT3 inhibitors in AML patients [10]. Although sequencing results from our cohort indicate that all FLT3^+^ patients harbored the wild-type sequence, a few cases of FLT3-ITD have been reported in CML patients [32]. Therefore, careful analysis of the mutational landscape of FLT3 in CML patients would suggest guidelines for more effective individualized combination therapies. In addition, the FLT3^+^ specimens were derived from both myeloid and lymphoid BP-CML patients, suggesting that both CML types could be susceptible to such combination therapies. Among the TKIs used in CML, ponatinib is a third generation BCR::ABL1 TKI designed to overcome TKI resistance caused by T315I mutation in BCR::ABL1. Since FLT3 is a known target of ponatinib, we investigated whether ponatinib alone was sufficient to achieve the therapeutic efficacy of the combination of BCR::ABL1 and FLT3 inhibitors. Based on our findings that single treatment with ponatinib, but not any of the first or second generation BCR::ABL1 inhibitors, could suppress FLT3 signaling, restore TKI sensitivity, and elicit marked cytotoxicity in BP-CML cells, we suggest a new drug application for ponatinib to treat FLT3^+^ CML patients. Together, we have established FLT3 as an effective prognostic marker and therapeutic target for patients in advanced phase CML as well as patients in CP progressing under TKI treatment. Since approved FLT3 inhibitors and detection methods are readily applicable, our results should help better predict and prevent BP progression.

Mechanistically, we found that FLT3 signaling leads to TKI resistance by triggering the aberrant induction of TAZ transcriptional coactivator. This was unexpected because TAZ is known to be hardly expressed in hematopoietic cells. Although the Hippo transducers TAZ and its paralog YAP are well-established oncogenes that activate TEAD-mediated transcription in solid tumor cells, YAP/TAZ were shown to be dispensable for or even act as tumor suppressor genes in the context of hematologic malignancies [29, 33]. In our study, we found TAZ and TEAD are critical effectors of FLT3 signaling in BP-CML cells. Our results establish two distinct regulatory modules for TAZ in CML. The 1) FLT3-JAK/STAT3-TAZ signaling pathway regulates the transcription of TAZ gene, and the 2) the Hippo pathway governs the post-translational regulation of TAZ protein. Therefore, in addition to repositioning FLT3 inhibitors as a means of blocking the transcription of TAZ, we propose a new clinical indication for YAP/TAZ-TEAD PPI (protein–protein interaction) inhibitors for the treatment of CML patients that could restore TKI sensitivity and impede BP progression by blocking TAZ nuclear translocation, TAZ-TEAD interaction, and the expression of TEAD target genes such as CD36.

How to treat patients in advanced phases of CML, and whether it is possible to prevent BP by optimizing drug treatment in CP is yet elusive. Here, we have established FLT3 as a prognostic marker and critical determinant for the acquisition of TKI resistance in advanced phase CML patients. Adding to the list of BCR::ABL1-dependent and -independent treatment modalities for BP-CML patients, we expect our discovery of novel therapeutic strategies targeting FLT3-JAK/STAT3-TAZ-TEAD-CD36 signaling to contribute to the effective prediction and treatment of CML.

## Conclusion

We report our discovery that aberrant FLT3 induction activates FLT3-JAK/STAT3-TAZ-TEAD-CD36 signaling pathway, which confers drug resistance to a wide range of BCR::ABL1 TKIs. Based on these results, we have demonstrated that co-administration of BCR::ABL1 inhibitors with recently developed FLT3 inhibitors, or single treatment with ponatinib, a third generation BCR::ABL1 TKI that targets BCR::ABL1 and FLT3 simultaneously, suppressed FLT3-TAZ signaling pathway, restored TKI sensitivity, and promoted cell death in drug resistant FLT3^+^ BP-CML cells. Together, our findings demonstrate the significance of FLT3 as a therapeutic target in BP progression and propose targeting FLT3 and downstream effectors are viable treatment strategies for preventing and overcoming TKI resistance in advanced phase CML.

### Supplementary Information

**Additional file 1:** **Supplementary Fig. S1. **Aberrant FLT3 expression in BP-CML cells promotes TKI resistance. **Supplementary Fig. S2. **Hippo transducers TAZ and TEAD mediate FLT3-induced drug resistance in BP-CML. **Supplementary Fig. S3. **FLT3-JAK-pSTAT3-TAZ signaling pathway in different cancer cell lines. **Supplementary Fig. S4. **Clinical evidence relating FLT3-TAZ signaling to BP-CML patients. **Supplementary Fig. S5. **FLT3-TAZ signaling promotes TKI resistance via CD36-mediated fatty acid uptake. **Supplementary Fig. S6. **Combined inhibition of BCR::ABL1 and FLT3 suppresses BP-CML tumor growth. **Supplementary Table 1. **AML driver genes listed in decreasing order of the ratio of their expression in BP-CML compared to CP-CML patient data acquired from NCBI Gene Expression Omnibus database (accession no. GSE4170). **Supplementary Table 2. **Clinical characteristics of BP-CML patients with distinct FLT3 expression patterns. **Supplementary Table 3. **BCR::ABL mutation status of BP-CML patients with distinct FLT3 expression patterns. 

## Data Availability

The RNA-seq data reported in this study are available in the Gene Expression Omnibus (GEO) database under GSE226360.

## Abbreviations

FLT3: Fms related receptor tyrosine kinase 3
TAZ: Transcriptional coactivator with PDZ-binding motif
TEAD: TEA domain transcription factor
CML: Chronic myeloid leukemia
AML: Acute myeloid leukemia
TKI: Tyrosine kinase inhibitor
BMMCs: Bone marrow mononuclear cells
CP: Chronic phase
AP: Accelerated phase
BP: Blast phase
ITD: Internal tandem duplication
TKD: Tyrosine kinase domain
scRNA-seq: Single-cell RNA sequencing
