# Supplementary material for: Targeting FLT3-TAZ signaling to suppress drug resistance in blast phase chronic myeloid leukemia
Source: Mol Cancer. 2023 Nov 6;22:177. doi: 10.1186/s12943-023-01837-4 (PMC10626670; doi:10.1186/s12943-023-01837-4)
Supplement: Supplementary file 1 — Additional file 1: Supplementary Fig. S1. Aberrant FLT3 expression in BP-CML cells promotes TKI resistance. Supplementary Fig. S2. Hippo transducers TAZ and TEAD mediate FLT3-induced drug resistance in BP-CML. Supplementary Fig. S3. FLT3-JAK-pSTAT3-TAZ signaling pathway in different cancer cell lines. Supplementary Fig. S4. Clinical evidence relating FLT3-TAZ signaling to BP-CML patients. Supplementary Fig. S5. FLT3-TAZ signaling promotes TKI resistance via CD36-mediated fatty acid uptake. Supplementary Fig. S6. Combined inhibition of BCR::ABL1 and FLT3 suppresses BP-CML tumor growth. Supplementary Table 1. AML driver genes listed in decreasing order of the ratio of their expression in BP-CML compared to CP-CML patient data acquired from NCBI Gene Expression Omnibus database (accession no. GSE4170). Supplementary Table 2. Clinical characteristics of BP-CML patients with distinct FLT3 expression patterns. Supplementary Table 3. BCR::ABL mutation status of BP-CML patients with distinct FLT3 expression patterns. [file 12943_2023_1837_MOESM1_ESM.docx]

**Supplementary Figures**

**
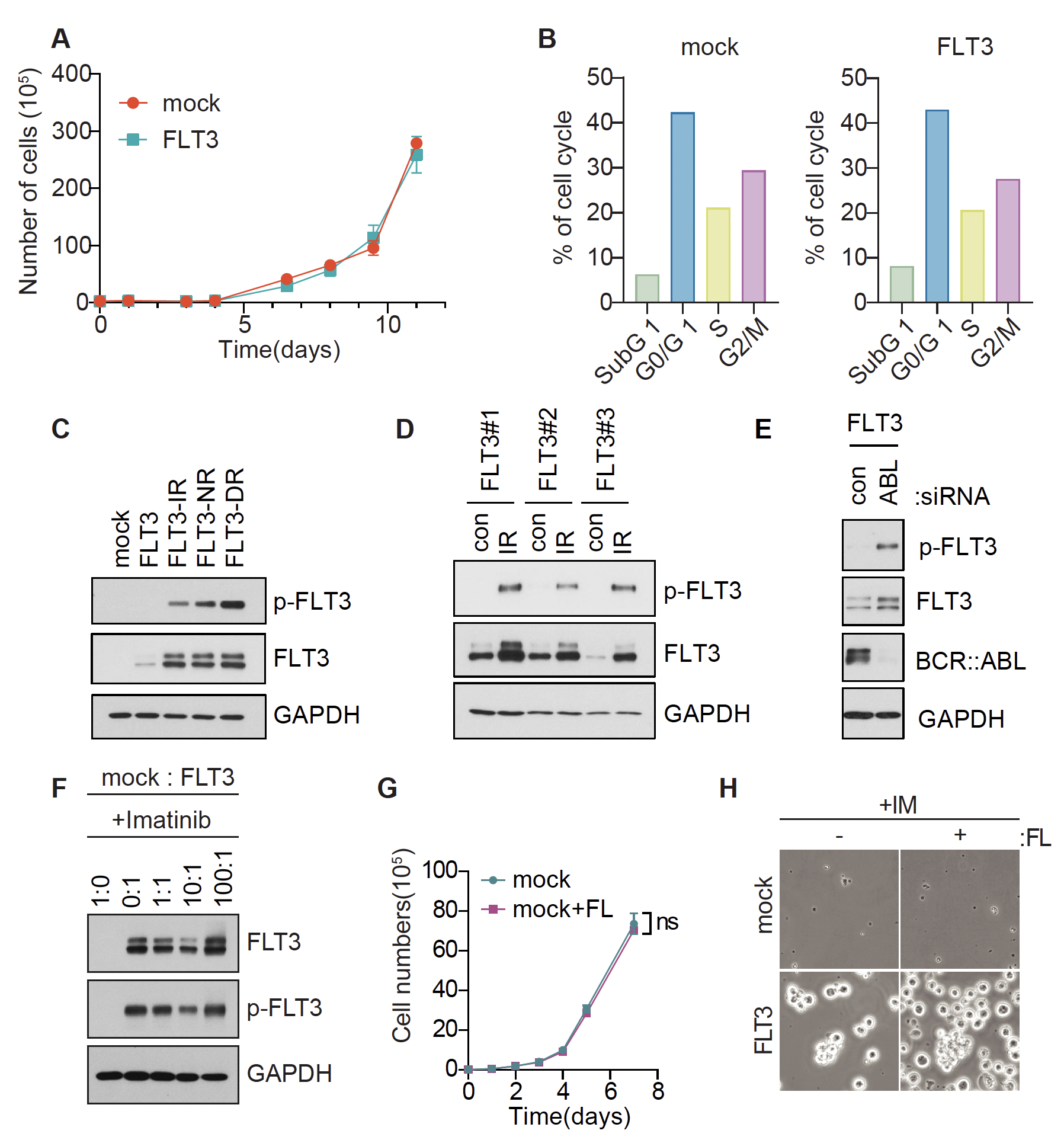
**

**Supplementary Fig. S1 Aberrant FLT3 expression in BP-CML cells promotes TKI resistance**

**A,** Measurement of cell growth in K562-mock and K562-FLT3 cells. Means of triplicates ± SD are shown.

**B,** Cell cycle distribution analysis of K562-mock and K562-FLT3 cells.

**C,** Immunoblotting analysis of phospho-FLT3 and FLT3 proteins in K562-mock, -FLT3, and TKI-resistant FLT3-IR, -NR, and -DR cells.

**D,** Immunoblotting analysis of FLT3 and phospho-FLT3 in control or imatinib-resistant K562-FLT3 clones.

**E,** Immunoblotting analysis of K562-FLT3 cells transfected with scrambled (con) or ABL siRNA for 48 hours after transfection.

**F,** Immunoblotting analysis of FLT3 expression after recovery in K562-FLT3 cells combined with control cells in different ratios in the presence of 1 μM imatinib.

**G,** Cell growth curve of K562-FLT3 cells treated with 20 ng/ml FLT3 ligand. All p-values were calculated using Student’s t-test and error bars are means of triplicates ± SD. A p-value of less than 0.05 indicates a statistical difference. ns, not significant

**H,** Cell images of K562-mock and K562-FLT3 cells subjected to 1 μM imatinib treatment with or without 20 ng/ml FLT3 ligand (FL) (Fig. 1K) at day 5.


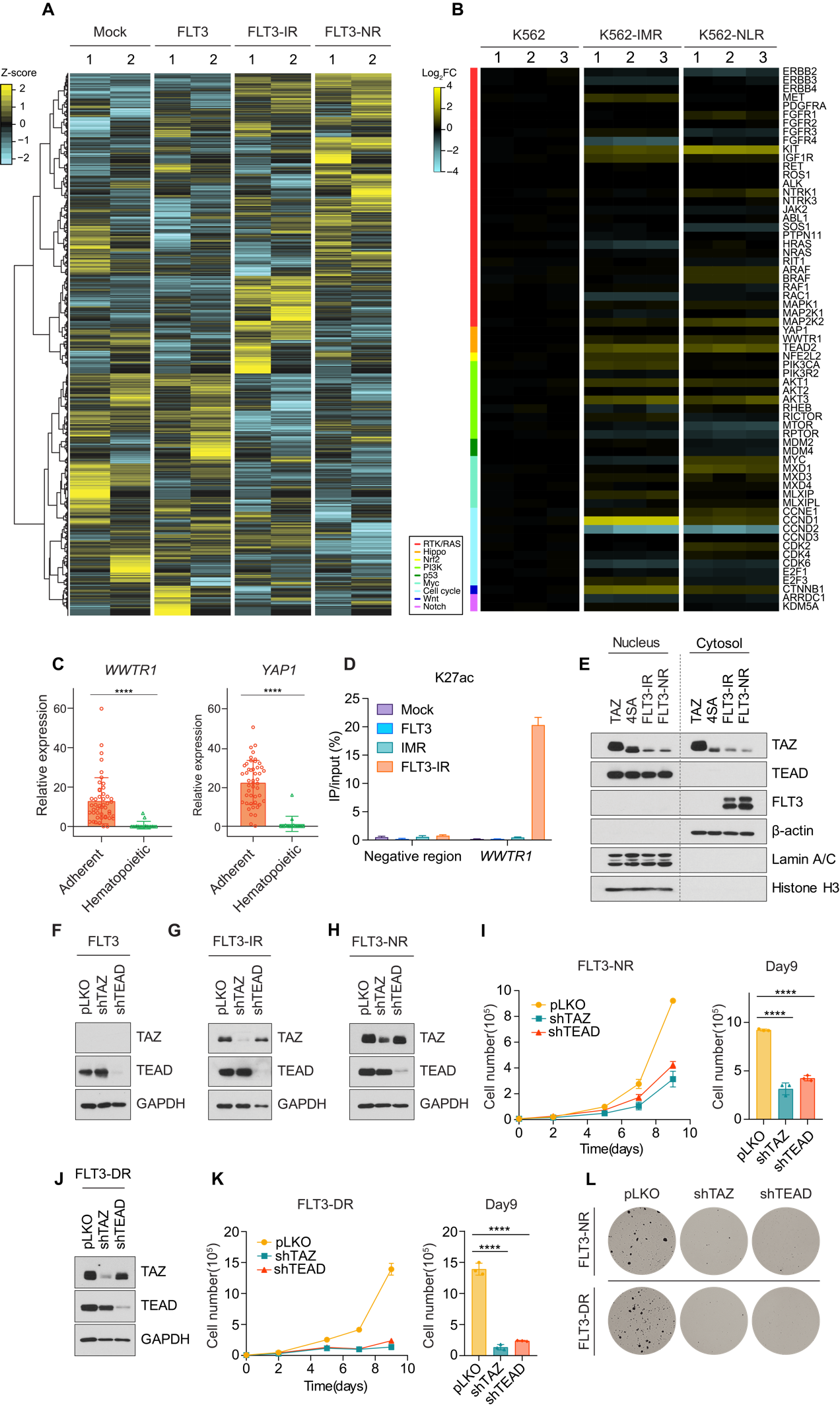


**Supplementary Fig. S2 Hippo transducers TAZ and TEAD mediate FLT3-induced drug resistance in BP-CML**

**A,** Heatmap analysis of RNA-seq data from K562-mock, FLT3, FLT3-IR, and -NR cells after batch correction of two independent trials. The numbers above each column denote the batch. The heatmap includes 12,000 highly expressed genes. Colors indicate the z-score of row-scaled gene expression.

**B,** Heatmap showing the relative expression of pro-tumorigenic factors within the major oncogenic pathways (color-coded as indicated in the legend) in RNA-seq data from K562, K562-IMR and K562-NLR cells. The color gradient reflects the log-fold change.

**C,** Relative WWTR1 and YAP1 transcript levels in adherent (n = 47) and hematopoietic (n = 17) cell lines. Data were obtained from the Human Protein Atlas database. p-values were calculated using Student’s t-tests and error bars are means ± SD are shown. ****p < 0.0001.

**D,** ChIP-qPCR showing the levels of K27ac at *WWTR1* locus of indicated cells.

**E,** Immunoblotting analysis of K562-TAZ, TAZ-4SA, FLT3-IR, and FLT3-NR cells subjected to nucleus-cytosol fractionation assay. Lamin A/C and histone H3 proteins were used as nuclear markers. β-actin was used as a cytosolic marker.

**F-H and J,** Immunoblotting analyses showing the efficiency of shRNA-mediated knockdown of TAZ or TEAD in K562-FLT3 (D), K562-FLT3-IR (E), K562-FLT3-NR (F), and K562-FLT3-DR (H) cells.

**I and K,** Cell growth curve for K562-FLT3-NR cells (G) and K562-FLT3-DR cells (I) transduced with shRNAs targeting TAZ or TEAD treated with 20 nM nilotinib or 1 μM dasatinib, respectively, for 9 days (left). Cells were counted on day 9 (right). p-values were calculated using one-way ANOVA and error bars are means of triplicates ± SD. ****p < 0.0001.

**L,** Colony formation assay performed on K562-FLT3-NR cells (upper row) and K562-FLT3-DR cells (lower row) transduced with shRNAs targeting TAZ or TEAD treated with 20 nM nilotinib or 1 μM dasatinib for 3 weeks.


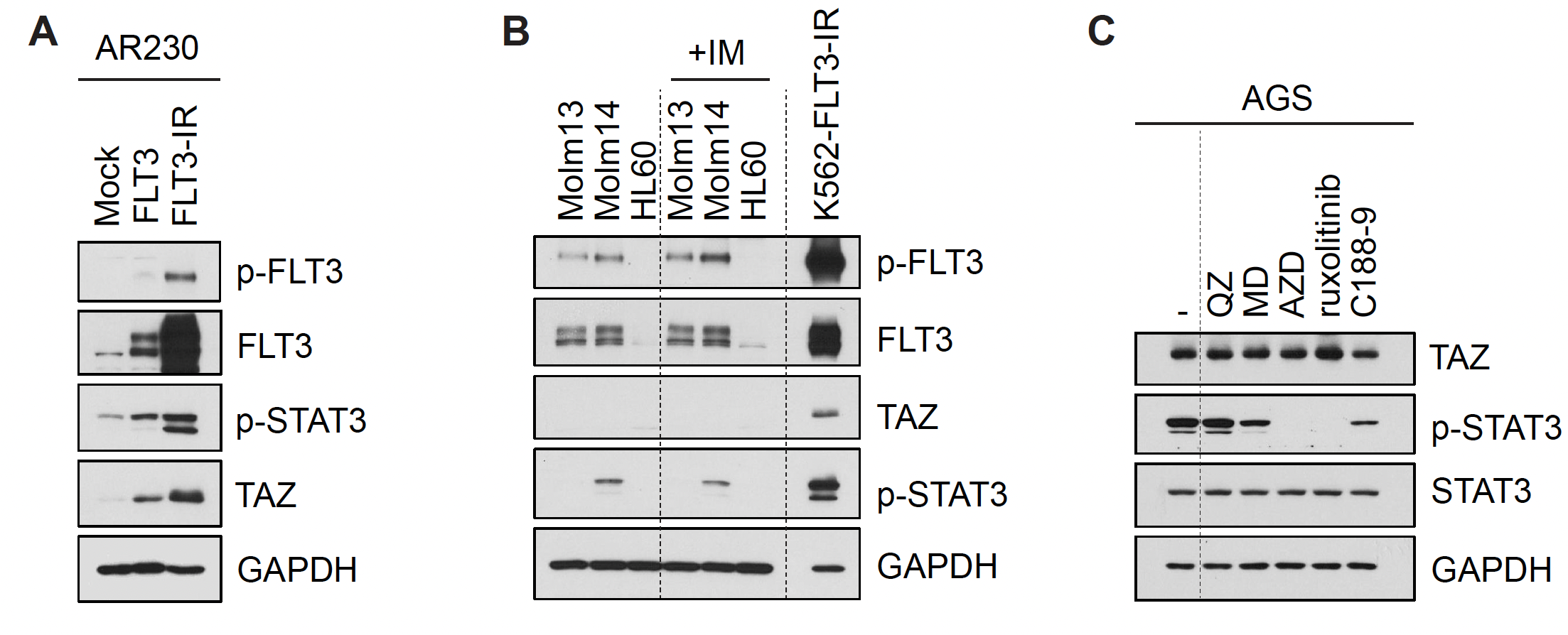


**Supplementary Fig. S3 FLT3-JAK-pSTAT3-TAZ signaling pathway in different cancer cell lines**

**A,** Immunoblotting analysis of FLT3 signaling in AR230-Mock, -FLT3, and -FLT3-IR cells.

**B,** Immunoblotting analysis of FLT3-pSTAT3-TAZ signaling components in AML cell lines (Molm13, Molm14, HL60) and K562-FLT3-IR cells before and after imatinib treatment.

**C,** Immunoblotting analysis of FLT3-pSTAT3-TAZ signaling components in AGS gastric cancer cells after treatments with 30 nM quizartinib (QZ), 0.3 μM, midostaurin (MD), 3 μM AZD-1480, 10 μM Ruxolitinib, and 30 μM C188-9 for 16 hrs.


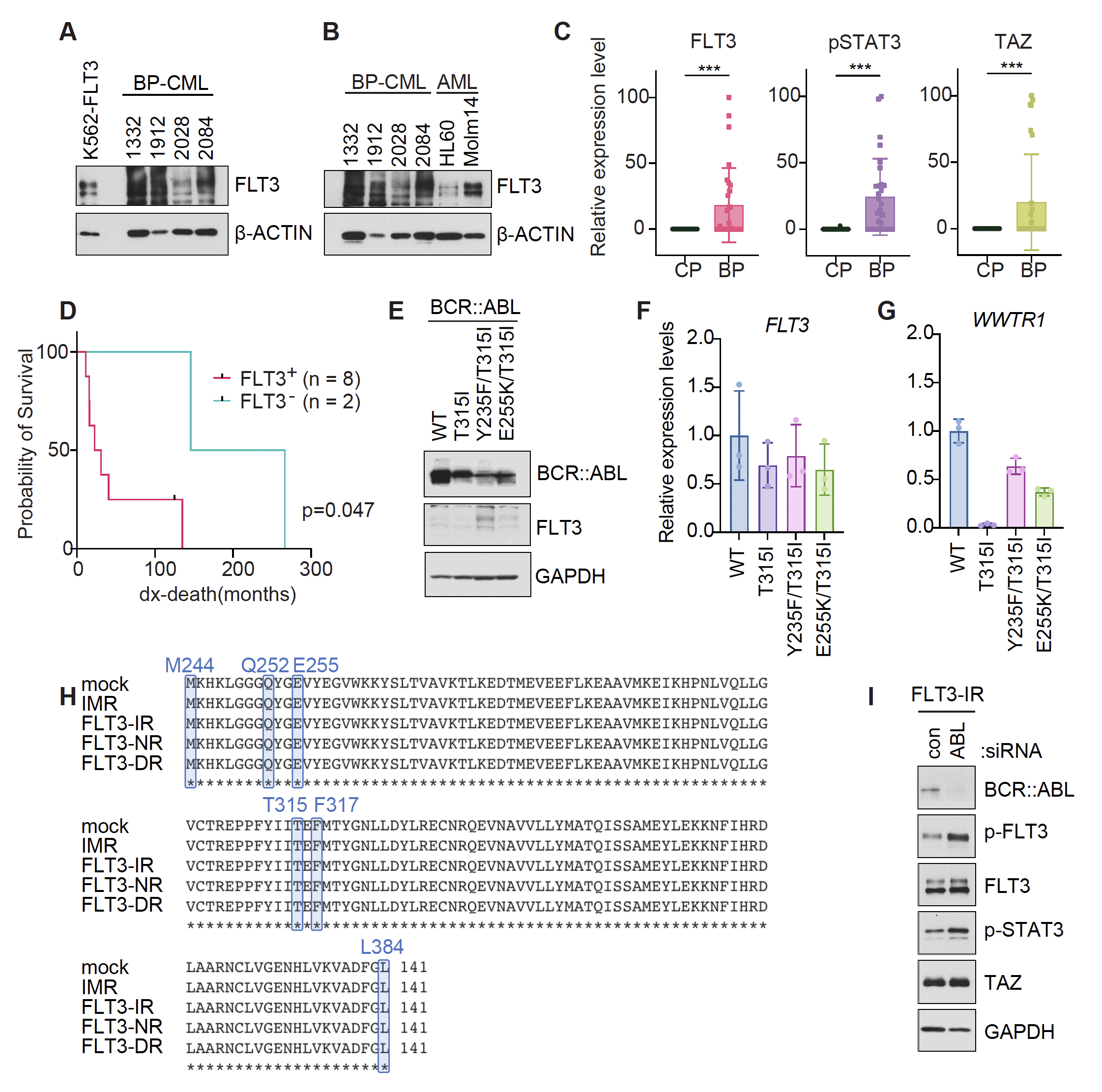


**Supplementary Fig. S4 Clinical evidence relating FLT3-TAZ signaling to BP-CML patients**

**A,** Immunoblotting analysis of FLT3 expression levels in FLT3-positive BP-CML patient samples (1332, 1912, 2028, 2084) and K562-FLT3 cells.

**B,** Immunoblotting analysis of FLT3 expression levels in FLT3-positive BP-CML patient samples (1332, 1912, 2028, 2084) and AML cell lines, HL60 (FLT3 wild type) and Molm14 (FLT3-ITD).

**C,** Quantitative analysis of FLT3, pSTAT3, and TAZ protein levels in clinical samples obtained from CP and BP-CML patients (n = 29 each). Relative immunoblot band intensities in Fig. 4A-C were quantified with the Image J program. ***p < 0.001. p-values were calculated using Student’s t-test and error bars are means ± SD.

**D,** Kaplan-Meier survival curves for FLT3^+^ and FLT3^-^ BP-CML patients from diagnosis to death (months). FLT3^+^ group (n = 8), FLT3^-^ group (n = 2). p = 0.047. p-values were calculated using Log-rank (Mantel-Cox) test.

**E,** Immunoblotting analysis of BCR::ABL1 and FLT3 in Ba/F3 cells expressing either BCR::ABL1-WT, T315I, Y253F/T315I, or E255 V/T315I mutants.

**F** **and** **G,** qPCR analyses of FLT3 (F) and TAZ (G) mRNA expression levels in Ba/F3 cells expressing either BCR::ABL1-WT, T315I, Y253F/T315I, or E255V/T315I mutants.

**H,** The alignment of the BCR::ABL1 amino acid sequence within recurrent BCR::ABL1 mutation regions in K562-Mock, -IMR, FLT3-IR, and FLT3-DR cells. Each point mutation was denoted by highlighting it in blue boxes within the sequence.

**I,** Immunoblotting analysis of FLT3-TAZ signaling in K562-FLT3-IR cells transfected with scrambled (con) or ABL siRNA for 48 hours.


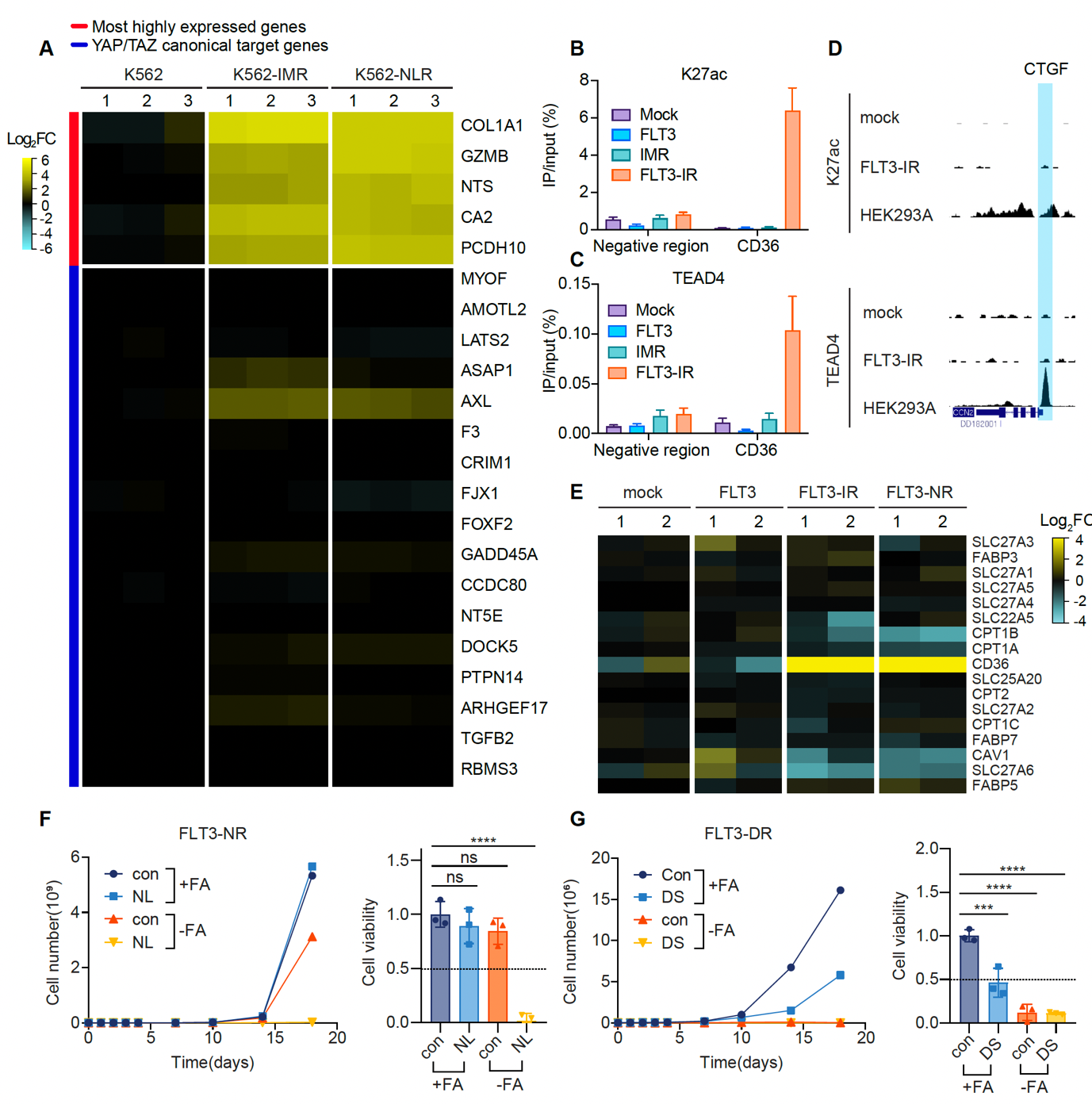


**Supplementary Fig. S5. FLT3-TAZ signaling promotes TKI resistance via CD36-mediated fatty acid uptake**

**A,** Heatmap analysis of RNA-seq data from K562, K562-IMR, and K562-NLR cell lines. The heatmap represents gene expression levels, with the most strongly enhanced genes labeled in red. Additionally, the canonical YAP/TAZ target genes are labeled in blue. Each row corresponds to a specific gene, while each column represents a different cell line. The color gradient reflects the relative log fold change, with brighter yellow colors indicating higher expression.

**B,** ChIP-qPCR showing the levels of K27ac at CD36 locus of indicated cells.

**C,** ChIP-qPCR showing the levels of TEAD4 that was bound to the CD36 locus of indicated cells.

**D,** ChIP-seq analysis of TEAD4 and H3K27ac enrichment at the CTGF promoter region (highlighted) in K562-mock, K562-FLT3-IR, and HEK293A cells.

**E,** Heatmap analysis of RNA-seq results from K562-mock, K562-FLT3, K562-FLT3-IR, and -NR cells showing the relative expression of fatty acid transport genes. n = 2 independent experiments.

**F and G,** Growth curve (left) and cell viability measured on day 18 (right) in K562-FLT3-NR (C) and K562-FLT3-DR (D) cells subjected to either normal medium (+FA) or fatty acid-free medium (-FA) with or without 20 nM nilotinib (NL) or 1 μM dasatinib (DS) treatment. p-values were calculated using one-way ANOVA with Bonferroni corrections for multiple comparisons and error bars are means of triplicate ± SD. ***p < 0.001, ****p < 0.0001; ns, not significant (p > 0.05).


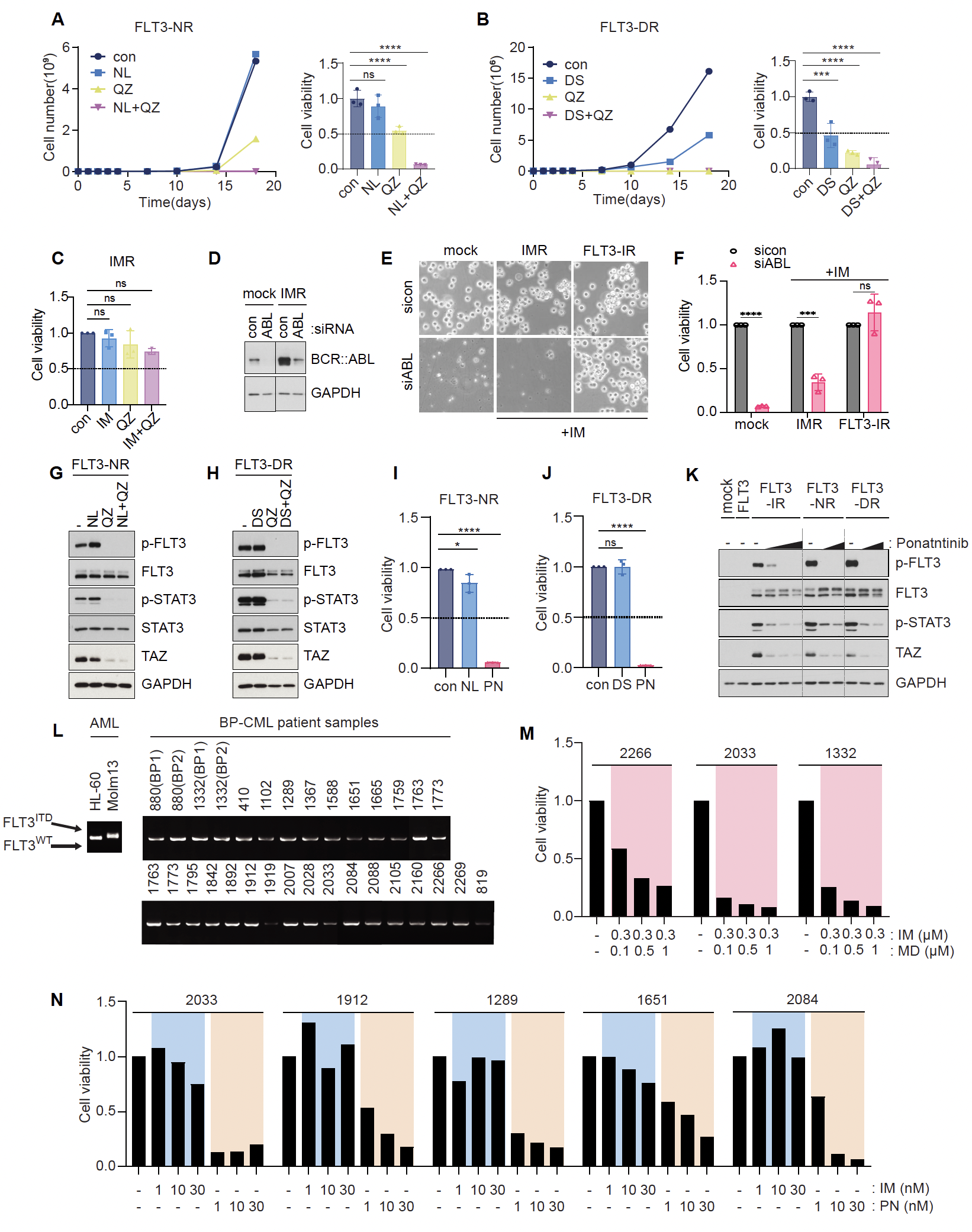


**Supplementary Fig. S5. Combined inhibition of BCR::ABL1 and FLT3 suppresses BP-CML tumor growth**

**A,** Growth curve (left) and cell viability (right) measured in TKI-resistant K562-FLT3-NR cells treated with 20 nM nilotinib (NL), 30 nM quizartinib (QZ), or the combination of nilotinib and quizartinib (NL+QZ) for 18 days. Cell viability of each group was measured on day 18 (right). **p < 0.01, ****p < 0.0001; ns, not significant (p > 0.05).

**B,** Growth curve (left) and cell viability (right) measured in TKI-resistant K562-FLT3-DR cells treated with 1 μM dasatinib (DS), 30 nM quizartinib (QZ), or the combination of dasatinib and quizartinib (DS+QZ) for 18 days. Cell viability of each group was measured on day 18 (right). **p < 0.01, ****p < 0.0001; ns, not significant (p > 0.05).

**C,** Cell viability measured in spontaneous imatinib resistant K562-IMR cells treated with 1 μM imatinib (IM), 30 nM quizartinib (QZ), or the combination of imatinib and quizartinib (IM+QZ) for 7 days. ns, not significant (p > 0.05).

**D,** Immunoblotting analysis of K562-Mock, IMR, FLT3-IR cells transfected 50nM siRNA of con (scrambled) or ABL for 48 hours.

**E and F,** Cell images (E) and cell viabilities (F) of K562-Mock, -IMR, FLT3-IR cells transfected with scrambled (con) or ABL siRNA for 72 hours. K562-IMR and -FLT3-IR cells were treated with 1μM imatinib. p-values were calculated using Multiple unpaired t-tests and error bars are means of triplicates ± SD. ***p < 0.001; ****p < 0.0001; ns, not significant.

**G,** Immunoblotting analysis of FLT3-pSTAT-TAZ signaling components in K562-FLT3-NR cells treated with 20 nM nilotinib (NL), 30 nM quizartinib (QZ), or the combination of nilotinib and quizartinib (NL+QZ) for 16 hrs.

**H,** Immunoblotting analysis of FLT3-pSTAT-TAZ signaling components in K562-FLT3-DR cells treated with 1 μM dasatinib (DS), 30 nM quizartinib (QZ), or the combination of dasatinib and quizartinib (DA+QZ) for 16 hrs.

**I,** MTT analysis showing the viability of K562-FLT3-NR cells treated with 20 nM nilotinib or 10 nM ponatinib for 5 days. *p < 0.05, ****p < 0.0001.

**J,** MTT analysis showing the viability of K562-FLT3-DR cells treated with 1 μM dasatinib or 10 nM ponatinib for 5 days. ****p < 0.0001; ns, not significant (p > 0.05).

**K,** Immunoblotting analysis of FLT3-pSTAT-TAZ signaling components in FLT3-mediated TKI-resistant cell lines treated with ponatinib (0, 1, 3, 10 nM in FLT3-IR; 0, 3, 10nM in FLT3-NR and -DR) for 16 hrs.

**L,** Sequencing analysis of BP-CML patient samples to verify recurrent FLT3 mutations in AML, including the ITD, D835Y, and F691L mutations. Images of gel electrophoresis and sequencing results indicate that BP-CML patients harbor wild type FLT3 sequences. AML cell lines HL-60 (FLT3 wild type) and Molm13 (FLT3-ITD) were used as negative and positive controls, respectively, to verify the band shift of FLT3-ITD mutation.

**M,** Individual cell viability measurements of BMMCs isolated from FLT3^+^ imatinib-resistant BP-CML patients treated with the combination of imatinib (IM) and midostaurin (MD) for 5 days.

**N,** Individual cell viability measurements of BMMCs isolated from FLT3^+^ imatinib-resistant BP-CML patients treated with either imatinib (IM) or ponatinib (PN) for 5 days.

**A-C, F, I-J,** p-values were calculated using one-way ANOVA with Bonferroni corrections for multiple comparisons. Error bars are means of triplicates ± SD. A p-value of less than 0.05 indicates a statistical difference.

**Supplementary Table 1.**

AML driver genes listed in decreasing order of the ratio of their expression in BP-CML compared to CP-CML patient data acquired from NCBI Gene Expression Omnibus database (accession no. GSE4170).

**Supplementary Table 2.**

Clinical characteristics of BP-CML patients with distinct FLT3 expression patterns.

**Supplementary Table 3.**

BCR::ABL mutation status of BP-CML patients with distinct FLT3 expression patterns.

**
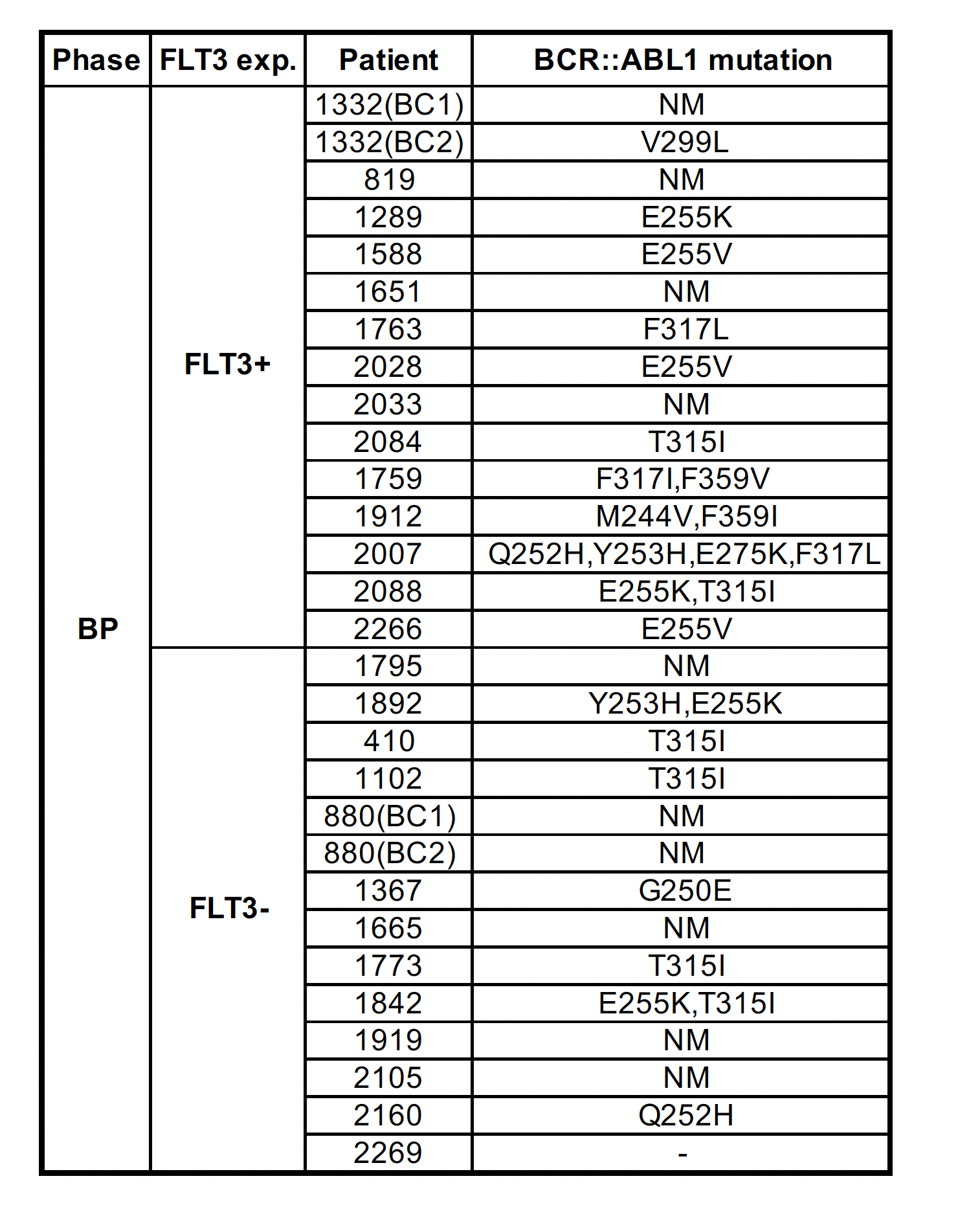
**

**Methods**

**Public data analysis**

The DNA microarray data of CML patients used in this study were retrieved from the Gene Expression Omnibus. The mRNA expression data of CML patients in the chronic phase (CP), accelerated phase (AP), and blast phase (BP) were obtained from GSE4170. YAP/TAZ transcript expression data were obtained from the Human Protein Atlas (HPA) database in the form of matrices that included normalized transcripts per million (nTPM) values. p-values were calculated using Prism 9.

**Cell culture, viral infection, and generation of TKI-resistance K562 cells**

K562 (ATCC, CCL-243) and K562-IMR-ATCC (spontaneous imatinib-resistant cell line), (ATCC, CCL-3344) cells were purchased from the American Type Culture Collection (ATCC). K562 cells were maintained in RPMI (Hyclone, SH30027.01), and HEK293T cells were maintained in DMEM (Hyclone, SH30022.01) containing 10% FBS (Gibco, 16000-044) and 1% Penicillin (Gibco, 15140-122) in a humidified incubator at 37°C with 5% CO2. For generating spontaneous imatinib-resistant K562 cells (K562-IMR), we treated imatinib by gradually increasing the dose from 0.2 μM to 1 μM during a 6 month period. For viral infection of K562 cells, HEK293T cells were transfected with retroviral MSCV vectors containing either FLT3 constructs, p-BABE vector containing TAZ, TAZ-4SA constructs, or a lentiviral pLKO vector containing shTAZ, shTEAD constructs using transfection Reagent (Polyplus) according to the manufacturer's protocol. For generating MSCV-FLT3 plasmid, we purchased FLT3-ITD vector (Addgene, #74499) and replaced FLT3-ITD to FLT3 wild type sequence by mutagenesis. 48 hrs after transfection, the retroviral/lentiviral supernatant was filtered through a 0.45 μm filter, supplemented with 10 μm/ml polybrene, and used to infect K562 cells. 48 hrs after infection, K562 cells were selected with puromycin (2 μm/ml). To generate TKI-resistant K562-FLT3-IR, -NR, and -DR cells, each group was treated with 1 μM imatinib, 20 nM nilotinib, and 1 μM dasatinib until the acquisition of drug resistance (about 17 days). To maintain TKI resistance, each cell line was further cultured in media containing each drug every 3–5 days.

**Chemical compounds**

The following chemical compounds were used in this study: Imatinib (Selleckchem, S24750), Dasatinib (Santacruz, sc-358114), Nilotinib (Selleckchem, S1033), Ponatinib (Selleckchem, S1490), Quizartinib (Selleckchem, S1526), Midostaurin (Cayman, 10459), AZD-1480 (Selleckchem, S2162), C188-9 (Selleckchem, S8605), BP-1-102 (Selleckchem, S7769), Ruxolitinib (Selleckchem, S1378) and FLT3 ligand (PeproTech, 30019).

**Cell growth analyses and cell viability assays**

For cell growth analysis, 0.1 - 2 x 10^5^ cells were plated per 12-well plate (Falcon, 353043) with media. Each well was resuspended by pipetting and trypan blue stained cells were counted by Countess (Thermo, A27977). Cells were replaced with fresh medium containing each drug every 3–5 days. For cell viability assay, cells from each well were precipitated and assessed with the MTT assay kit (Roche, 11465007001) or the Cell Titer Glo assay kit (Promega, G9242) according to the manufacturer´s recommendations.

**Flow cytometry for cell cycle analysis and dilution assays**

For each cell cycle analysis, 10^6^ cells were harvested and FxCycle™ PI/RNase Staining Solution (Thermo, F10797) was used according to the manufacturer’s protocol and added to the flow cytometer (Thermo, Attune NxT). For dilution analyses, K562-mock and K562-FLT3 cells were mixed in different ratios (1:1, 1:10, 1:100). A total of 2x10^5^ cells were treated with 1 μM imatinib for 39 days and fresh medium containing the drug was replaced every 3–5 days. On day 39, the cells were fixed in 4% paraformaldehyde (Thermo, 28908)/PBS for 15 min and then permeabilized with 0.1% Triton-X/PBS for 10 min. The cells were washed and blocked with 3% BSA/PBS for 30 minutes and then incubated for 1 hour at RT with primary antibodies diluted in 0.5% BSA/PBS followed by a 30-min incubation with secondary antibodies diluted in 0.5% BSA/PBS. Finally the cells were resuspended in PBS and analyzed by flow cytometry (Thermo, Attune NxT).

**Immunoprecipitation assay**

1x10^7^ cells were rinsed once with PBS, they were in lysed with ice-cold lysis buffer (0.15 M NaCl, 0.05 M Tris-HCl, 0.5% Triton X-100), and one tablet each of EDTA-free protease/phosphatase inhibitor cocktail (Thermo, 78446). For immunoprecipitations, magnetic beads (Bio-rad, 1614023) and TEAD antibody (Santa Cruz, sc-101184) were added to the lysates and incubated overnight at 4°C. Then, the samples were washed with lysis buffer, denatured with the addition of 2X sample buffer, boiled for 8 min and subjected to immunoblotting analysis.

**Immunofluorescence microscopy**

Cells were seeded onto 24-well plates on coverslips (Everest, SB-Shifix25) and incubated for 30 min. The cells were fixed with 4% paraformaldehyde (Thermo, 28908) in PBS for 20 min and then permeabilized with 0.1% Triton-X/PBS for 10 min. After cells were washed and blocked with 3% BSA/PBS for 30 minutes, they were incubated overnight at 4°C with primary antibodies diluted in 3% BSA/PBS. Secondary antibodies were diluted in 3% BSA/PBS. Then, the cells were incubated for 2 hrs with secondary antibodies diluted in 3% BSA/PBS. Finally, the slides were mounted with Prolong gold antifade reagent containing DAPI (Invitrogen, P36930), and fluorescent images were acquired using a confocal microscope.

**RNA extraction, cDNA synthesis, and quantitative real-time PCR analysis.**

After harvesting 2 x 10^5^ cells for RNA extraction using the RNeasy Plus mini kit (Qiagen, 4134), the resulting RNA samples were reverse transcribed to complementary DNA using iScript reverse transcriptase (Bio-Rad, 1708890). Then, qRT-PCR was performed using the KAPA SYBR FAST Kapa SYBR FAST qPCR Master Mix (KM4103) and the 4376600 real-time PCR system (Applied Biosystems). The following primers were used for qPCR : FLT3, 5’- AAGCAATTTAGGTATGAAAGCCAGC-3’ (forward) and 5’-CTTTCAGCATTTTGACGGCAACC-3’ (reverse); YAP, 5’- CCTTCTTCAAGCCGCCGGAG-3’ (forward) and 5’-CAGTGTCCCAGGAGAAACAGC-3’ (reverse); TAZ, 5’-AATGGAGGGCCATATCATTCGAG-3’ (forward) and 5’-GTCCTGCGTTTTCTCCTGTATC-3’ (reverse); CD36, 5’-CAGGTCAACCTATTGGTCAAGCCT-3’ (forward) and 5’- GCCTTCTCATCACCAATGGTCC-3’ (reverse); GAPDH, 5’-GCAAATTCCATGGCACCGT-3’ (forward) and 5’-TCGCCCCACTTGATTTTGG-3’ (reverse); HPRT, 5’-AGAATGTCTTGATTGTGGAAGA-3’ (forward) and 5’-ACCTTGACCATCTTTGGATTA-3’ (reverse) for human samples. mFLT3, 5’- GTGCTGACGTTTGAAGACCTCC-3’ (forward) and 5’- GGTGACCAACACATTCCTGGCT-3’ (reverse); mHPRT, 5’-GCAGTACAGCCCCAAAATGG-3’ (forward) and 5’-ACAAAGTCCGGCCTGTATCCAA-3’ (reverse) for mouse samples.

**Immunoblotting analysis**

After precipitating cells by centrifugation at 3000 g for 3 min and lysing them in SDS sample buffer, the resulting protein samples were loaded onto the wells of a 9% SDS-page gel and transferred to PVDF membranes (Millipore). The membranes were blocked with 5% skim milk in TBS-T buffer (tris-buffered saline with 0.2% Tween-20) for 1 hr at room temperature before incubation with the corresponding primary antibodies overnight at 4˚C with gentle agitation. Then, after the membranes were washed 3 times with TBS-T buffer, they were incubated with secondary antibody in 5% skim milk/TBS-T. After the membranes were washed 4 times, they were developed using X-ray films.

The antibodies used in this study are as follows: Vinculin (Santacruz, sc-7364), BCR::ABL1 (CST, 2862), CD36 (CST, 14347), GAPDH (Santacruz, sc-25778), FLT3 (CST, 3462), p-FLT3 Y842 (CST, 4577), p-FLT3 Y591 (CST, 3474), p-STAT3 (CST, 9145), p-STAT5 (CST, 9351), p-STAT1(CST, 9167), STAT1 (CST, 9172), STAT5 (CST, 25656), pan-TEAD (CST, 13295), STAT3 (CST, 4904), TAZ (CST, 83669), YAP (63.7) (Santcruz, sc-101199), YAP/TAZ (CST, 8418), LATS (CST, 3477), PARP (CST, 9532), p-BCR::ABL1 Y412 (CST, 2865), p-BCR::ABL1 Y245 (CST, 2861), p-BCR Y177 (CST, 3901), p-ERK (CST, 4377), ERK 1/2 (CST, 4695), p-S6K (CST, 9234), p-Src (CST, 2101), p-AKT (CST, 4060), p-P65 (CST, 3033), β-Actin (Santacruz, sc-47778), MEK1/2 (CST, 8727), Histone H3 (CST, 4499), and Lamin A/C (CST, 4777).

**Colony formation assay**

Each well of a 6-well plate (Falcon, 353046) was coated with 1.5 ml of bottom agar (RPMI containing 10% FBS and 0.8% low melting agarose (Sigma, A9414). Then, 1–2 x 10^4^ cells were suspended in 1.5 ml of top agar (RPMI containing 10% FBS and 0.4% low melting agarose) and plated into each well. The cells were incubated for approximately three weeks. Fresh media exchanges containing each drug were performed every three days. Colonies were stained using 0.005% crystal violet and the colonies per well were counted.

**siRNA transfection**

K562-FLT3-IR cells were transfected with transfection mix (100 μl of Opti-MEM™ medium (ThermoFisher, #31985070), 12 μl RNAi Max (Invitrogen, 13778150), and 2 μl of 20 μM siRNA) for 48 hrs. The Neon Transfection System (ThermoFisher, MPK5000) was used to electroporate the K562-mock, -IMR, and -FLT3-IR cells for the purpose of introducing siRNA molecules (ABL siRNA). The conditions of electroporation for this study: 1450V, 3 pulse, 10ms, 50 nM DNA, and 1x10^5^ cells. The siRNA used in this study are as follows: control siRNA (Dharmacon siGENOME non-targeting control pool #D-001206-13-200) and siLATS1/2 (Dharmacon siGENOME SMARTPool #M-004632-00-0005 and #M-003865-02-0005), and siABL (Dharmacon, #L-003100-00-0010).

**Metabolites profiling using UPLC-Orbitrap-MS**

**Sample preparation**

After adding 100 μL of ice-cold 70% methanol containing an internal standard (reserpine at a final concentration of 2 ppm) to the cell pellets (3 x 10^6^), the solution was vortexed for 30 sec. Then, liquid nitrogen was used to perform three successive freeze/thaw cycles to lyse the cells. The cell lysates were centrifuged for 10 min at 14,000 rpm. The supernatants were immediately used in metabolomic experiments. An equal volume of each sample was pooled to create a quality control (QC) sample. A normalization of the DNA was then performed on the lysates and the concentrations were measured with a Nano-MD UV-Vis spectrophotometer (Scinco, Seoul, Korea).

**Instrumental conditions**

An Acquity® UPLC HSS T3 column (1.8 μm particle size, 2.1 mm x 100 mm, Waters, USA) was used to perform liquid chromatography on an Ultimate 3000 UHPLC system (Thermo Scientific, Jose, CA, USA) at 40°C. Mobile phase A (0.1% formic acid in distilled water) and mobile phase B (0.1% formic acid in methanol) were combined and set to flow at 0.4 mL·min-1 for the gradient elution. The sample analysis was performed using a gradient program as follows: the initial conditions, 99% A and 1% B (v/v), were maintained for 1 min, and then a linear gradient was initiated that reached 20% B over 2 min. After an increase to 70% mobile phase B for 4 min, it was slowly increased to 100% over 6 min and maintained at 100% for 2.5 min. The column was re-equilibrated to the initial conditions within 1.5 min and stabilized for 2 min. The injection volume was 10 μL and all samples were maintained at 5°C during the analysis. Prior to running the sample sequence, the QC sample was examined for conditioning. It was also examined in the first, middle, and final samples of the batch to assess instrument consistency. An LTQ Orbitrap Velos Pro™ system mass spectrometer (Thermo Scientific, San Jose, CA, USA) equipped with a heated electrospray ionization source (HESI) in positive and negative ionization mode was used for the detection of metabolites. The HESI parameters were as follows: heater temperature, 200°C; sheath gas flowrate, 35 arb (arbitrary units); auxiliary gas flow rate, 5 arb; sweep gas flowrate, 10 arb; capillary temperature, 320°C; and S-lens RF level, 67.5%. Data acquisition was performed at a resolution of 60,000 in the centroid mode using a mass range of m/z 50–1000. The Xcalibur 2.2 (Thermo Scientific, San Jose, CA, USA) software system was used for data acquisition and processing.

**Statistical analysis and metabolite identification**

Peak alignments and data collection were carried out using Xcalibur 2.2 and Compound Discoverer 2.1 (Thermo Fisher Scientific, San Jose, CA, USA). Prior to statistical analysis, the compound intensities were normalized to internal standard areas. SIMCA 14.1 (Umetrics, Inc., Ume, Sweden) and Metabolanalyst (http://www.metaboanalyst.ca/) were used to perform the multivariate analysis. Meanwhile, the variable importance in the projection (VIP) values that suggested greater discriminatory power for each metabolite were taken as the coefficients for peak selection. The Student’s t-test was used to identify statistically significant metabolites among the different groups. The HMDB, Metlin, and Lipidblast databases were used to perform putative identification of the metabolites via MS/MS fragmentation matching

**RNA sequencing analysis**

For the RNA-seq experiment, two independent experiments were performed in triplicate and pooled for RNA extraction. Raw single-strand sequencing data were aligned with the Salmon package and mapped to the human GRCh38 reference genome. Ensembl gene IDs were annotated with their respective gene symbols using the bioMart package. Reads per kilobase of transcript per million mapped reads (RPKM) were calculated for each gene. Batch correction was done using ‘removeBatchEffect’ function in the Limma R package. PCA analysis was performed using the plotMDS package, and analysis of differentially expressed genes was done using the Limma R package. The RNA-seq data reported in this study are available in the Gene Expression Omnibus (GEO) database under GSE226360.

**Public RNA sequencing data analysis**

To investigate differences based on FLT3 induction, we performed a bulk RNA-seq data analysis using the Limma R package. This analysis was independent of the experimental data analysis and focused specifically on comparing gene expression profiles between samples with FLT3 induction and those without. For this analysis, we utilized public bulk RNA-seq data (Accession no. GSE4170) as the control group. The same analysis pipeline based on the Limma R package was applied to compare the gene expression profiles between the FLT3 induced K562 cells with WT K562 cells.

**Chromatin immunoprecipitation (ChIP) assay**

K562 or HEK293A cells were cross-linked for 15 min with 1% formaldehyde and then quenched with 0.125 M glycine for 10 min at room temperature. 5 × 10^6^ cells were used for H3K27ac ChIP, and 3 × 10^7^ cells were used for TEAD4 ChIP. Based on a size of 5 × 10^6^ cells, the cell pellets were lysed with 200 μl of cell lysis buffer (10 mM Tris-Cl pH 8.0, 10 mM NaCl, 0.2% NP-40) with protease inhibitor (Roche, 11697498001) and 1 mM DTT for 10 min on ice. The nuclei were isolated by centrifugation at 7,400 rpm for 30 seconds and gently resuspended in 200 μl of nuclear lysis buffer (50 mM Tris-Cl pH 8.0, 10 mM EDTA, 1% SDS) with protease inhibitor and 1 mM DTT. The lysates were sonicated for 10 cycles (30 seconds on/30 seconds off) and then incubated for 1 hour with 10 μg rabbit IgG and 10 μl of Protein A magnetic beads (Invitrogen, 10001) for pre-clearing. The immunoprecipitation was conducted with 10 μl of Protein A magnetic beads and 1 μg of the H3K27ac antibody (Abcam, ab4729) or 200 μl of TEAD4 antibody (Santa Cruz, sc-101184) overnight at 4°C on a rotator. The next day, the immunocomplexes were washed with IP Wash I Buffer, High salt buffer, IP Wash II buffer, and a final wash with TE buffer (pH 8.0). The washed immunocomplexes were eluted with 200 μl of elution buffer (1% SDS and 0.1 M NaHCO3) for 1 hour at 45°C on a thermomixer at 1,000 rpm. The eluates were de-crosslinked with the addition of 0.25 M NaCl and RNase A (1 μg/μl) and then incubated overnight in a 65°C water bath. The next day, after a 2-hr incubation with 4 μl of Proteinase K (NEB, P8107S), the ChIP DNA was purified with a QIAquick PCR purification kit (QIAGEN, 28106). 2 μl of 1:10 diluted ChIP DNA was subjected to ChIP-qPCR with 1 μl 10 μM primer and SYBR Green Master Mix. The negative region primer set is designed to amplify regions without ChIP-seq signals in each gene region. The following primers were used for ChIP-qPCR : hWWTR1 Negative region, 5’-GATGGTCAACTTTGGGGCAA-3’ (forward) and 5’-TGTTGCTTTCCACATTGCCA-3’ (reverse); hWWTR1 K27ac region, 5’-CCGCTCAGACCTGCATCT-3’ (forward) and 5’-TCAGGCCACTTTCCCTTTGA-3’ (reverse); hCD36 Negative region, 5’-TGAGTGAGAACATGCGGAGT-3’ (forward) and 5’-TGAAGCTGGAAACCGTCATTC-3’ (reverse); hCD36 K27ac region, 5’-CTGTCATTGGTGCTGTCCTG-3’ (forward) and 5’-TCTTCTGGATAAGCAGGTCTCC-3’ (reverse); hCD36 TEAD4 BS, 5’-CAACCCACATTCTGTTCGCA-3’ (forward) and 5’-TCTCTTCCCTTGTCTCAGCA-3’ (reverse).

**ChIP-seq library construction**

ChIP-seq libraries were constructed using 40 μl of purified ChIP DNA and a NEXTflexTM ChIP-seq kit (PerkinElmer, NOVA-5143-02) according to the manufacturer's instructions. ChIP DNA was end-repaired and size-selected using AMPure XP beads (Beckman, A63881). Other procedures from adenylation to PCR amplification were done according to standard ChIP-seq library construction protocols. The quality of the resulting ChIP-seq libraries was determined using a Bioanalyzer and the High Sensitivity chip (Agilent). The average size of the ChIP-seq libraries ranged from 250 to 350 bp. For multiplexing, equimolar quantities of each library were combined while considering the sequencing depth per sample (20 to 40 million reads per library). The ChIP-seq libraries were sequenced using an Illumina NextSeq platform with single-end reads of 76 bases.

**Bioinformatic analyses of ChIP-seq**

ChIP-seq analyses were performed as previously described (Roe et al., 2017). For alignment of ChIP-seq reads, raw reads were mapped to the reference human (hg19) genome assemblies using Bowtie2, and duplicated reads were removed using SAMtools. The makeBigWig tool (in the HOMER suite) was used to generate bigWig files for visualization with the UCSC genome browser.

**Patient samples**

A total of 81 CML samples (39 CP including NEL and CHR samples, 3 AP, and 39 BP) from bone marrow (BM) and peripheral blood (PB) were obtained from 49 CML patients. Among them, 8 patients had serial samples. Mononuclear cells (MCs) were isolated by Ficoll-Paque (GE Healthcare) density gradient centrifugation. Samples were frozen in 10% dimethyl sulfoxide (DMSO; Sigma-Aldrich) in fetal bovine serum (Sigma-Aldrich), stored in liquid nitrogen, and later thawed for analysis. All human samples were obtained from the Korea Leukemia Bank and the protocol was approved by the Institutional Review Board. Patient consent was obtained in accordance with the Declaration of Helsinki. For immunofluorescence, immunoblotting, qPCR, and cell viability assays of the patient cells, we thawed the cell stocks and centrifuged them at 800g for 5min to remove their supernatants. Every experiment was finished within 7 days to ensure proper cell viability. To verify recurrent FLT3 mutations, patient cDNA samples were amplified and sequenced with FLT3 primers (F, 5’- GCAATTTAGGTATGAAAGCCAGC - 3’; R, 5’- TTTTACAGGCAGACGGGCAT - 3’).

**Animal experiments**

NOD/SCID mice were purchased from Koatech. For tumor xenograft models, K562-FLT3-IR cells (5 x 10^6^) in 1/3 matrigel (BD biosciences, 354248) were injected subcutaneously into 6-week-old male mice. Ten mice were assigned to each group. Four days after injection, imatinib (100 mg/kg), quizartinib (30 mg/kg), ponatinib (30 mg/kg), or vehicle (22% hydroxypropyl-β-cyclodextrin for quizartinib and 0.5% sodium carboxymethyl cellulose for imatinib and ponatinib) was administered 6 days per week by oral gavage. Tumor height and width were measured with calipers to calculate tumor volume (= width^2^ x height/2). Mice were sacrificed 10 weeks after the experiment began. All animal experiments were approved by the Yonsei University Institutional Animal Care and Use Committee (Documentation #201610-435-02).

**Statistics and reproducibility**

All quantitative data were obtained from at least three independent biological replicates. Data are presented as means ± standard deviation (s.d.) unless otherwise noted in the figure legends. Statistical differences between two groups were examined using two-tailed, unpaired or paired Student's t-tests and one-way analyses of variance (ANOVA) with Bonferroni corrections for multiple comparisons. Statistical tests were performed using the GraphPad Prism 9.0 software (GraphPad Software, CA, USA). Two-sided p-values of less than 0.05 were considered significant.
